# Evidence from genome wide association studies implicates reduced control of Epstein-Barr virus infection in multiple sclerosis susceptibility

**DOI:** 10.1186/s13073-019-0640-z

**Published:** 2019-04-30

**Authors:** Ali Afrasiabi, Grant P. Parnell, Nicole Fewings, Stephen D. Schibeci, Monica A. Basuki, Ramya Chandramohan, Yuan Zhou, Bruce Taylor, David A. Brown, Sanjay Swaminathan, Fiona C. McKay, Graeme J. Stewart, David R. Booth

**Affiliations:** 1 Centre for Immunology and Allergy Research, Westmead Institute for Medical Research, University of Sydney, Sydney, Australia; 2 Menzies Research Institute Tasmania, University of Tasmania, Hobart, Australia

**Keywords:** Multiple sclerosis, Genetics, Epstein-Barr virus, Risk genes, Expression quantitative trait loci, miRNA, Transcription factors

## Abstract

**Background:**

Genome wide association studies have identified > 200 susceptibility loci accounting for much of the heritability of multiple sclerosis (MS). Epstein-Barr virus (EBV), a memory B cell tropic virus, has been identified as necessary but not sufficient for development of MS. The molecular and immunological basis for this has not been established. Infected B cell proliferation is driven by signalling through the EBV produced cell surface protein LMP1, a homologue of the MS risk gene CD40.

**Methods:**

We have investigated transcriptomes of B cells and EBV-infected B cells at Latency III (LCLs) and identified MS risk genes with altered expression on infection and with expression levels associated with the MS risk genotype (LCLeQTLs). The association of LCLeQTL genomic burden with EBV phenotypes in vitro and in vivo was examined. The risk genotype effect on LCL proliferation with CD40 stimulation was assessed.

**Results:**

These LCLeQTL MS risk SNP:gene pairs (47 identified) were over-represented in genes dysregulated between B and LCLs (*p* < 1.53 × 10^−4^), and as target loci of the EBV transcription factor EBNA2 (*p* < 3.17 × 10^−16^). Overall genetic burden of LCLeQTLs was associated with some EBV phenotypes but not others. Stimulation of the CD40 pathway by CD40L reduced LCL proliferation (*p* < 0.001), dependent on CD40 and TRAF3 MS risk genotypes. Both CD40 and TRAF3 risk SNPs are in binding sites for the EBV transcription factor EBNA2, with expression of each correlated with EBNA2 expression dependent on genotype.

**Conclusions:**

These data indicate targeting EBV may be of therapeutic benefit in MS.

**Electronic supplementary material:**

The online version of this article (10.1186/s13073-019-0640-z) contains supplementary material, which is available to authorized users.

## Background

Epstein-Barr virus (EBV), a memory B cell tropic virus, has been identified as necessary but not sufficient for development of MS [1]. This implies it is necessary to initiate pathogenesis, but the molecular and immunological basis for this has not been established. Its potential importance in pathogenesis has been highlighted by the success of therapies removing memory B cells (antiCD20 monoclonal antibodies) and the failure of therapies which increase them (antiTACI monoclonal antibodies) [2]. Causality is also indicated from three large studies using different approaches. From a large longitudinal study, adults without EBV antibodies who later developed MS also were infected by EBV [3]. The risk of developing MS for those with the HLA-DRB1*1501 allele and for EBV infection is higher for the combination than due to each factor independently, suggesting an interaction and causality [4, 5]. Finally, the time to developing MS is shorter for those with late EBV infection than for those with early EBV infection, consistent with infection affecting development of disease [6].

First infection with EBV at a later age confers greater risk of developing MS than early infection [7]. In addition, association studies indicate higher EBV loads for those with MS compared to controls [8] and decreased T cell response to EBV antigen on therapy in those with a clinical response, potentially reducing epitope spreading and autoinflammation [9], suggesting an effect on disease progression. Due to its ability to immortalise B cells, EBV can cause a number of malignancies [10], notably nasopharyngeal cancer and Hodgkin’s lymphoma. In immunocompromised individuals, such as those receiving transplants, unrestrained EBV infection can be fatal. MS risk SNPs are over-represented as target sites for the EBV transcription factor EBNA2 [11], especially in conjunction with the vitamin D receptor binding sites [12]. Collectively, these data indicate this near-ubiquitous virus is usually controlled by a sustained immune response and are consistent with the hypothesis that failure of EBV control can induce disease, including MS. Moreover, EBV control may continue to be impaired and worsen in established MS. With increasing disease duration, EBV-specific T cells progressively decline, consistent with T cell exhaustion and inversely proportional to anti-EBNA-1 titres [13]. There is a higher incidence of spontaneous transforming events in long-term culture of MS B cells [14], together supporting the existence of a higher proportion of latently EBV-infected B cells in MS.

Where EBV causes tumours, symptoms of infection, or uncontrolled lymphocyte proliferation, possible treatments include antiCD20 antibodies to remove the host target cell, anti EBV T cells from tissue banks, or genetic manipulation of anti EBV T cells [15]. Vaccines to specifically boost immune response are in development [15]. To promote further development of these approaches and to identify novel ones, a better understanding of immune evasion by EBV is needed. The genetic variation that increases risk of MS may indicate molecular pathways controlling EBV and so targets for improved therapy.

More than 200 gene loci have been associated with MS using genome wide association studies (GWAS) [16]. Their effect is mainly on gene expression. EBV has four transcriptomes corresponding to three latency phases and a lytic phase. Latency III phase is the major target of the four phases for the immune response in chronic disease [17]. To find risk genes potentially affecting control of EBV infection, firstly, we identified MS risk genes expressed differently in B cells and EBV-infected B cells at Latency III (called LCLs, for lymphoblastoid cell lines) using RNAseq. Second, we used in silico data on the association of genotypes with expression in whole blood and LCLs, to identify LCLeQTLs. We then retested the SNP associations with expression in independent cohorts of LCLs and B cells. Further, we sought evidence of EBV regulation of these LCLeQTLs using pathway analysis and analysis of EBV transcription factor binding sites near LCLeQTLs. Lastly, we assessed the effect of genetic burden of LCLeQTLs on EBV phenotypes in vitro and in vivo.

Many of the LCLeQTL genes mapped on to the LMP1/LMP2 signalling pathway. For one of the first genes to be identified as an MS risk factor using GWAS, CD40 [18], a T cell activation gene, the protective allele was shown to have higher expression [19]. This is counter-intuitive, since T cell activation is thought to promote MS risk. A potential explanation for this paradox is that higher expression of CD40 reduces signalling through the EBV protein LMP1, a homologue of CD40, inhibiting EBV survival in B cells. This could occur via competition for intracellular signalling molecules shared between these pathways. Increased cross-linking of CD40 has been shown to inhibit LCL proliferation [20, 21]. Here we have investigated CD40L inhibition of LCL proliferation and the effect of the CD40 risk genotype and that of another MS risk gene, TRAF3, a ligand for both CD40 and LMP1.

These data provide genetic support for a facilitative role of EBV infection in MS, but do not prove it. They indicate molecular processes important in regulating LCL proliferation, and so molecular targets for control of EBV infection and potentially reducing MS progression.

## Methods

### Samples

All blood samples were collected from healthy controls with informed consent (Westmead Hospital Human Research Ethics Committee Approval 1425). B lymphocytes were purified by immunomagnetic human B cell enrichment Kit (Stem Cell Technologies) according to the manufacturer’s instructions. For LCL generation, fresh or frozen PBMCs from the B cell donors were infected with supernatant from B95.8 producer cell line for 1 h at 37 °C. Cells were then suspended in complete medium, consisting of RPMI-1640 (Lonza) supplemented with 10% fetal bovine serum (FBS, Sigma Aldrich), 2 mM l-glutamine (Life Technologies), 50 units per ml penicillin/50 g per ml streptomycin (Life Technologies) plus 2 μg/ml of cyclosporin A (Sigma Aldrich) and plated at 2.5 × 10^6^ or 5 × 10^6^ cells/well in 48-well plates. Cells were fed weekly, expanded into 25-cm^2^ flask. LCLs were cryopreserved in 10% DMSO (MP Biomedical) 50% FBS and RPMI.

### RNAseq

Global gene expression profiling using RNAseq was carried out for *n* = 5 LCL and CD19+ B cells (matching donors). Total RNA was first isolated using the RNeasy Mini Kit (Qiagen) before RNAseq library preparation using the TruSeqV2 Library Preparation kit (Illumina). The indexed libraries were pooled 10 plex, and 50 bp single-end reads were sequenced on the HiSeq 2500 (Illumina). Reads were assessed for quality using FastQC, aligned to hg19 using TopHat2 [22], and summarised to RPKM gene level expression using SAMmate [23]. An average of 18 million mapped reads was obtained for each sample at an overall alignment rate of 80.5%. Differentially expressed genes were calculated using EdgeR [24]. A cut-off of 1% false discovery rate was selected to select differentially expressed genes, see Additional file 1: Table S1. The raw and processed sequencing data generated have been submitted to the NCBI Gene Expression Omnibus (GEO; http://www.ncbi.nlm.nih.gov/geo/) under accession number GSE126379 [25].

### GTEx eQTL in LCL and WB

The rAggr website (www.raggr.usc.edu) was used to prepare the variant call format (VCF) for all 201 MS risk SNPs from rs ID and genomic coordinates (187 SNPs were located). eQTL data for 186 of these SNPs was available from the GTEx dataset [26] for both LCL and whole blood (WB). We filtered proximal genes from all eQTL data associated with MS risk SNPs in LCL and WB (Additional file 2: Figure S1) and merged the two datasets as a final SNP:gene table to compare the genotype effect of each MS risk SNPs in LCL and WB. The effect of risk allele relative to protective allele based on the slope data was then extracted. We identified the SNP:gene eQTL pairs with higher association in LCL than WB based on the *p* value being less than 0.05 in LCL, and of a lower rank in *p* value ranking system for SNP:gene list in LCL than WB. Also, of the genes with a *p* < 0.05 in LCLs, those with a reverse slope between LCLs and WB were included. The set of MS risk SNPs, all eQTL data for MS risk SNPs in LCL and WB, eQTL data for proximal genes of MS risk SNPs and SNP:gene pairs eQTL with higher association in LCL than WB are reported in Additional file 1: Table S2–S6, and the detailed workflow is in Additional file 2: Figure S2.

### Gene expression analysis of activated B cells, B cells and LCLs

The normalizied microarray gene expression data for LCL, activated B cells and B cells (*n* = 3 matching donors) were obtained from [27, 28]. Differentially expressed genes for activated B cell vs B cell and LCL vs B cell were calculated using the GEO2R web tool on NCBI Gene Expression Omnibus (GEO; http://www.ncbi.nlm.nih.gov/geo/).

### Pathway analysis

MetaCore by Clarivate Analytics (https://clarivate.com/products/metacore) was used to undertake pathway analysis of proximal genes for: (I) EBV transcription factors binding to MS risk SNPs, (II) SNP:gene eQTL pairs with higher association in LCL rather than WB, (III) all proximal SNP:gene eQTL pairs in LCL, all proximal SNP:gene eQTL pairs in WB and (IV) all proximal SNP:gene eQTL pairs in WB without SNP:gene eQTL pairs with higher association in LCL rather than WB.

### EBV transcription factors/MS risk SNPs

EBV transcription factors binding peak ChIP-seq data were extracted from the Regulatory Element Local Intersection (RELI) tool [11, 29]. Genomic coordinates of ChIP-seq data for BZLF1, EBNA2, EBNA3c, EBNA1 and EBNALP EBV transcription factors in LCL were prepared as a BED file. Bedtools v2.26.0 software was used to identify the overlap between MS risk SNPs, SNPs in LD with them, and EBV transcription factor binding peaks (Additional file 1: Table S7). All steps of this workflow are illustrated in Additional file 2: Figure S3.

### Genetic burden and EBV phenotypes in vitro and in vivo

The genetic burden (total amount of risk alleles) of MS risk SNPs was calculated by assuming 2 score for risk genotype, 1 score for heterozygous genotype and 0 score for protective genotype and summing up the scores of all MS risk SNPs for each LCL sample. Genotype data for MS risk SNPs was extracted for 176 CEU individuals from 1000 Genome and HAPMAP 3 projects LCL samples [30]. Intrinsic growth rate was available for LCLs from 92 CEU individuals [31]. The gene expression data for EBV from the same LCL samples was available on the EBV Portal [32, 33]. Also, estimated EBV DNA copy number in the same LCL samples was obtained [34]. Spearman’s rank correlation coefficient was used for testing the significance of correlations. Linear regression was used for testing the significance of correlation between genetic burden and EBV phenotypic elements (EBV genes, EBV DNA copy number and intrinsic growth rate). We also tested if the genetic burden of the LCLeQTL SNPs was associated with EBNA-1 IgG titres in data collated from a large anti-EBNA1 GWAS [35] using a linear mixed-effects kinship model fit by maximum likelihood.

### Genotyping and gene expression

In the Westmead LCL cohort, CD40 (SNP rs1883832) and TRAF3 (SNPs rs12588969, rs12148050) were genotyped using Taqman Assays, and gene expression was assayed using Taqman probes Hs00912657_m1, Hs00936778_m1 respectively (Life Technologies). Splicing of CD40 was determined as previously described [36].

### CD40 proliferation

For proliferation readout for the Westmead LCL cohort, LCLs were first labelled with Cell Trace Violet (CTV, Life Technologies) at a final concentration of 5 mM. 5 × 10^4^ LCLs were cultured with or without CD40L (250 ng/mL, Adipogen) in 5-mL polystyrene tubes (Becton Dickinson, BD) for 5 days. Day 5 cells were harvested and ran on FACSCantoII flow cytometer (BD). Median fluorescence intensity (MFI) of CTV was analysed using FlowJo.

### Statistics

Analysis of gene expression differences in the WIMR LCL and B cell cohort was conducted using GraphPad Prism 7 (GraphPad Software, USA) using paired and unpaired (where appropriate) two-tailed *T* tests to compare between groups. *P* values for overlaps were calculated using the hypergeometric distribution over-representation test [37].

## Results

### The LCL transcriptome

**Fig. 1:**
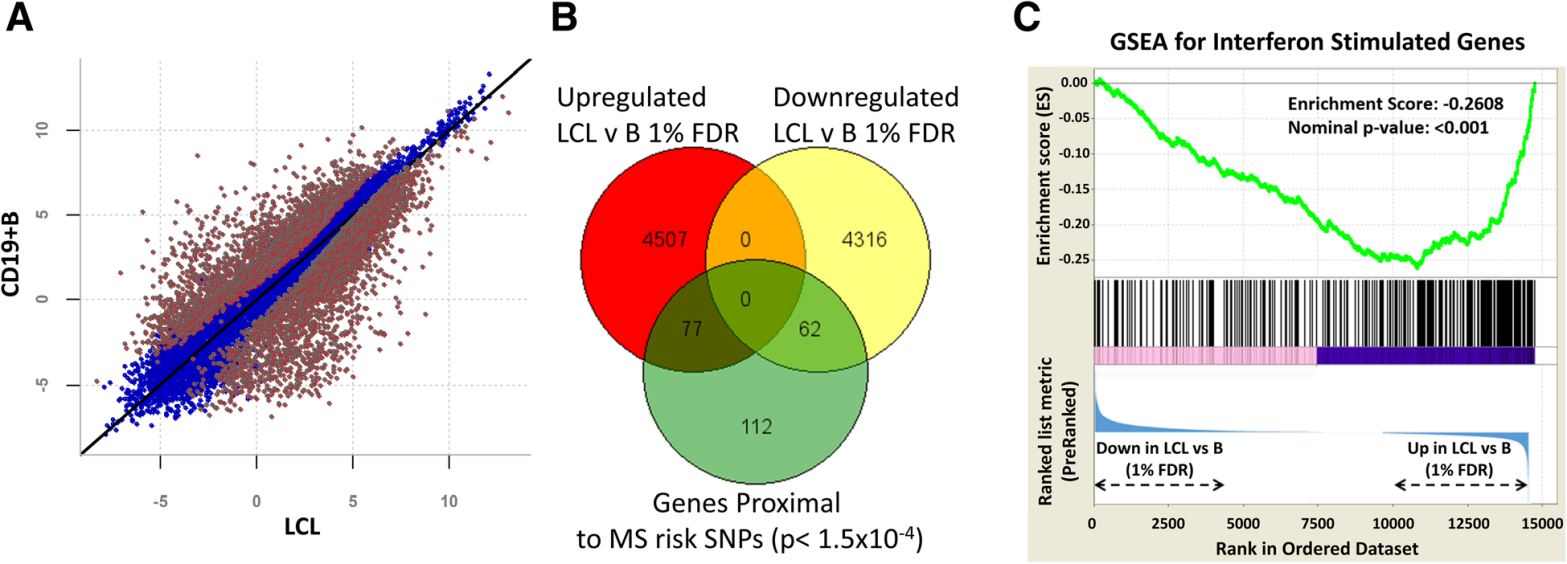
RNAseq reveals vast gene expression changes in LCLs compared to CD19+B cells. **a** Scatter plot highlighting genes differentially expressed at 1% FDR (red), **b** Genes proximal to MS risk SNPs are over-represented in the differentially expressed genes (hypergeometric over-representation test), **c** Gene Set Enrichment Analysis (GSEA) shows interferon stimulated genes are over-represented in genes upregulated in LCLs

To identify MS risk genes likely to contribute to variation in regulation of EBV infection, we first screened for risk genes with altered regulation in EBV-infected B cells (LCLs) compared to B cells. We used RNAseq to interrogate expression in ex vivo CD19+ B cells and in LCLs derived from them using EBV strain B95.8 infection. Consistent with their different phenotypes, the transcriptomes were very different between infected and uninfected B cells. At a false discovery rate (FDR) of 0.01, 8962 genes were expressed differently (Fig. 1) (Additional file 1: Table S1). Differentially expressed genes were enriched for interferon stimulated genes (*p* < 0.001, Fig. 1c).

### MS risk genes that are LCL eQTLs

**Fig. 2:**
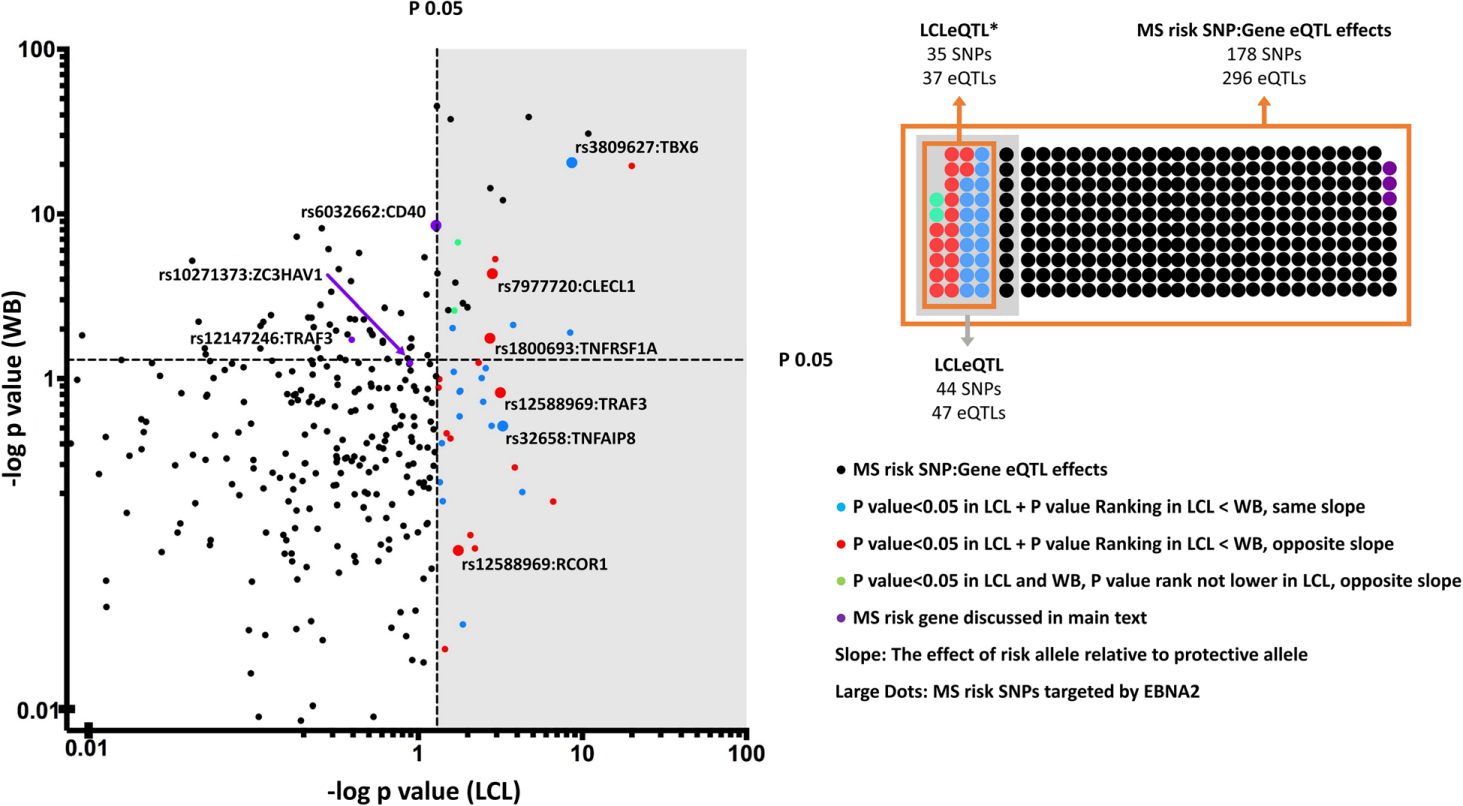
The effect of MS risk SNP genotype on expression of proximal genes in whole blood and LCLs. The GTEx eQTL dataset was first filtered for MS risk SNPs, and SNP:gene pairs were then plotted for effect of genotype (restricted to the genes closest to the MS risk SNPs—the proximal genes). SNP:gene pairs that were more strongly associated with expression in LCLs (coloured blue) and those with a different risk allele effect in LCLs compared to whole blood, more significant in LCLs (red) or opposite slope in LCLs(green) were identified as LCLeQTL*

**Fig. 3:**
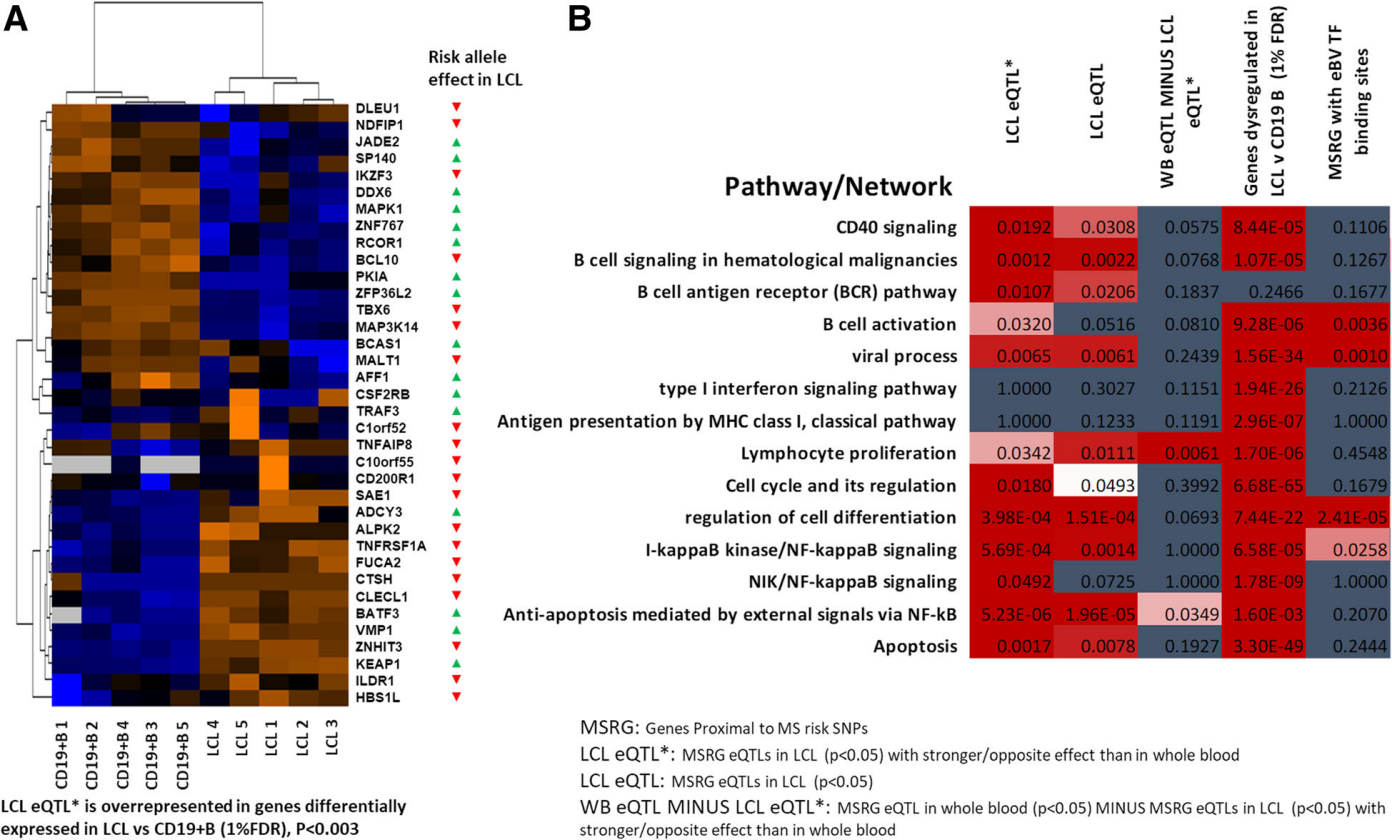
Genes and pathways with a stronger association of expression in LCLs compared to whole blood. **a** Heat map showing expression level of genes corresponding to the SNP:gene pairs that were more strongly associated with expression in LCLs compared to whole blood (LCLeQTL*) in CD19^+^ B cells and LCLs. **b** Over-representation of pathways and networks in LCLeQTL*(MSRG eQTLs in LCL (*p* < 0.05) with stronger/opposite effect than in whole blood); LCLeQTL(MSRG eQTLs in LCL (*p* < 0.05)); WB eQTL minus LCLeQTL*(MSRG eQTL in whole blood (*p* < 0.05) minus MSRG eQTLs in LCL (*p* < 0.05) with stronger/opposite effect than in whole blood); genes dysregulated in LCLs vs CD19^+^ B cells at 1% FDR; MSRG with EBV transcription factor binding sites; MSRG, genes proximal to MS risk SNPs. TF, transcription factor. *P* values were calculated using MetaCore (Clarivate Analytics)

Since risk SNPs mainly affects pathogenesis by altering gene expression [16], we identified SNPs associated with gene expression (expression quantitative trait loci; eQTLs) in LCLs. We identified that of 255 MS risk SNP:gene pairs, 47 were eQTLs in LCLs at *p* < 0.05 in the GTEx dataset. This list of 47 SNP:gene pairs which contains 44 SNPs and 47 genes we call LCLeQTL (Additional file 1: Table S2). Using GTEx data as a discovery dataset, and the RTeQTL data [38] as a replication set, of the 47 GTEx LCLeQTL SNP:gene pairs, 31 were tested in at least one of the RTeQTL cohorts, and 28 of these were replicated at *p* < 0.05, mostly with much lower *p* values (Additional file 1: Table S3). We then identified those 47 GTEx LCLeQTL SNP:gene pairs more associated with expression in LCLs than whole blood, as this would favour LCL expression as driving the pathogenic basis for their association with MS, more than expression in the immune cells of the blood (Fig. 2). As the statistical power for the whole blood cohort was greater, we based this comparison on rank of *p* value, rather than raw *p* values. Of these, 18 had the same genotype effect on expression in LCLs and whole blood, 17 of these had opposite genotype associations with expression. Finally, two did not have lower *p* values in LCLs, but are included in the list as having opposite genotype effects between LCLs and whole blood. This list of 37 SNP:gene pairs which contains 35 SNPs and 37 genes, we call the LCLeQTL* (Additional file 1: Table S4). Thirty-three of 47 LCLeQTL genes were in the genes differentially expressed between B cells and LCLs (over-representation *p* = 1.53 × 10^−4^), and 24/37 of the LCLeQTL* genes (*p* < 0.003, Fig. 3). Of the WBeQTLs (MS risk SNP:gene pairs in eQTL in whole blood at *p* < 0.05) (Additional file 1: Table S5), a lower proportion were in the differentially expressed gene list (35/57, *p* < 0.004), even less with the LCLeQTL* genes removed from the WBeQTL list (Additional file 1: Table S6, 28/47, *p* < 0.017). Of the 255 MS risk genes, 139 were differentially expressed between LCL and B cells (*p* < 1.44 × 10^−4^).

Despite these over-representations of LCLeQTL, LCLeQTL*, WBeQTL SNPs and especially MS risk genes in the genes differentially expressed between EBV-infected and B cells, it remains possible that the enrichment may point to the B cell functions of these genes/SNPs rather than viral contribution to pathogenesis. We compared genes differently expressed between B cells and activated B cells at FDR of 0.001 from published data [27]. Of the 1474 genes differentially expressed, 38 were MS risk genes (over-representation *p* value 2.47 × 10^−6^), 6 were LCLeQTL genes (over-representation p value 0.12) and 6 were LCLeQTL* genes (over-representation p value 0.046). However, 1992 genes were differentially expressed at this FDR between B cells and LCLs, 49 were MS risk genes (over-representation *p* value 2.59 × 10^−7^), 11 were LCLeQTL genes (over-representation *p* value 0.0046) and 9 were LCLeQTL* genes (over-representation p value 0.0074). This suggests the MS risk genes, particularly LCLeQTL* genes, are more dysregulated in LCLs than activated B cells compared to B cells, implicating their role in EBV infection is more important than in B cell activation.

### MS risk genes potentially regulated by EBV transcription factors

Harley et al. [11] and Ricigliano et al. [12] have identified an excess of EBNA2 binding sites among risk SNPs for MS and other autoimmune diseases. To identify EBV-specific enrichment, we sought risk SNPs/genes co-located with EBV transcription factor binding peaks with the expanded set of MS risk SNPs [16]. Of the 45,498 SNPs with listed associations in the GWAS catalogue, 871 were co-located with an EBV transcription factor (TF) binding site [11]. MS risk SNPs were co-located (28 of 201 mapped) with EBV TF binding peaks at an extraordinary level of enrichment (Additional file 1: Table S7, *p* = 3.17 × 10^−16^). Of the 44 LCLeQTL SNPs, 6 were co-located with EBNA2 peaks (*p* = 1.83 × 10^−4^). Of these 6 SNPs, 5 SNPs were also in the 35 LCLeQTL* SNP list (*p* = 5.1 × 10^−4^). The proximal genes to these 5 SNPs were TRAF3, RCOR1, TBX6, TNFAIP8, TNFRSF1A and CLECL1.

### MS risk genes on the LMP1/LMP2 Signalling pathways

**Fig. 4:**
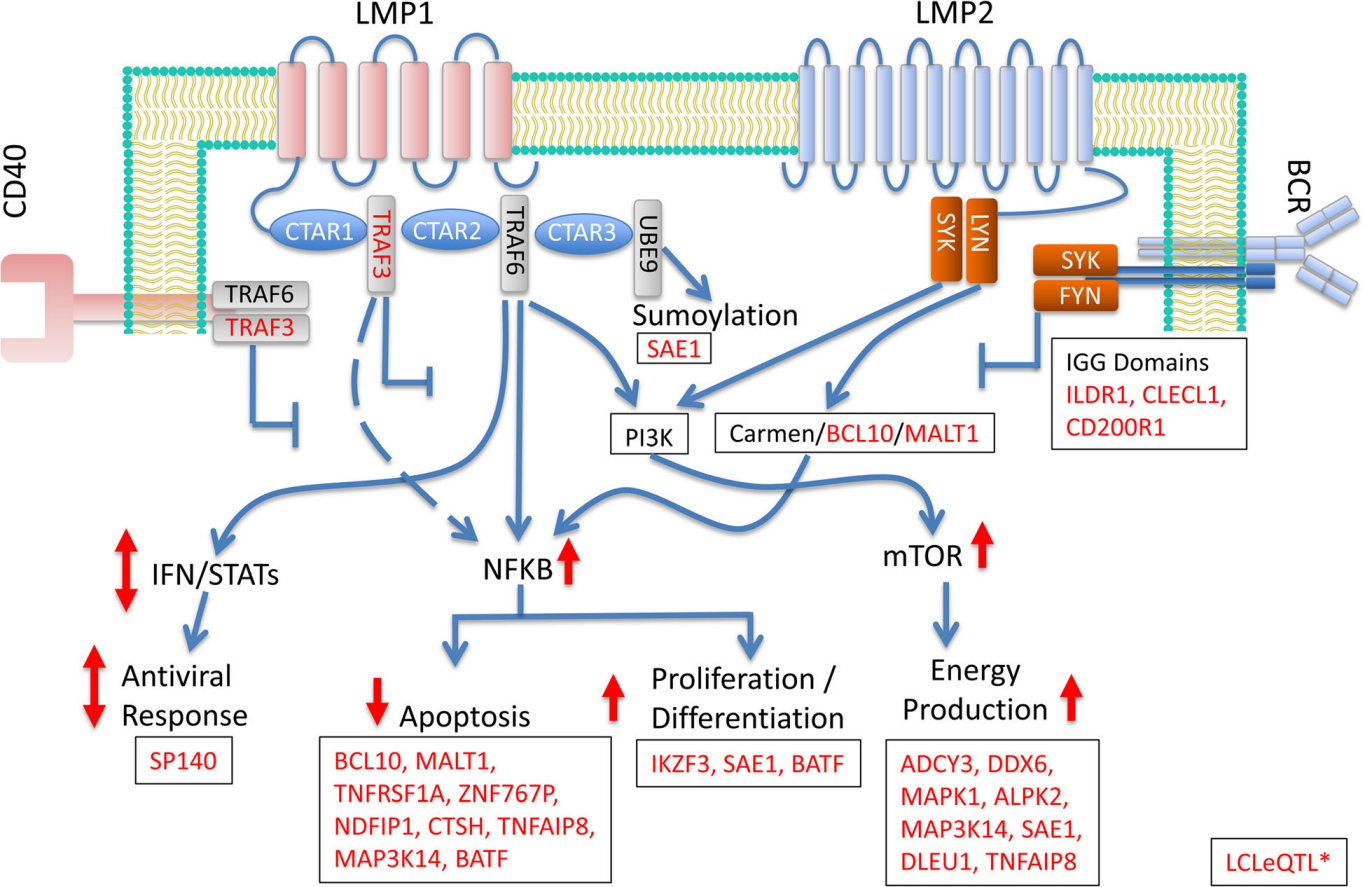
Model of LCLeQTL* gene roles in Latency III signalling pathways. Signalling from EBV proteins LMP1 and LMP2 leads to upregulation of NFKB, sumoylation, and mTOR1 pathways and altered IFN regulation. LCLeQTL* genes with roles on these pathways are in red. Signalling from the LMP1 homologue and MS risk gene CD40 inhibits LMP1 function (see Fig. 1). For similar reasons, signalling through the BCR, in this model, blocks LMP2 pathways. Arrows indicate proposed effect of signalling through LMP1/LMP2 on each of the downstream processes

Next, we mapped the LCLeQTL* genes on to the LMP1 and LMP2 signalling pathways (Fig. 4). The LMP1 pathway was as defined previously [39], which overlaps the CD40 pathway as described [40]. This pathway was confirmed and extended in the work of Li et al. [41], who used nasopharyngeal tumour mutations, a semi-agnostic approach, to define the pathway. The LMP2 pathway was as defined in Cen and Longnecker [42], who described its overlap with the B cell receptor (BCR) signalling pathway. LCLeQTL* gene-encoded protein TRAF3 binds directly to LMP1 and CD40; IgG domain containing proteins (genes CLECL1, CD200R and IL1DR) may bind or compete with LMP2/BCR. Molecules one signalling node down from this affect sumoylation (SAE1) and NFKB activation via the BCR (MALT1 and BCL10). NFKB is activated by both LMP1 and LMP2 pathways and, in turn, regulates genes controlling apoptosis (different roles for BCL10 and MALT1 than above; TNFRSF1, ZNF767P, NDFIPP1, CTSH, TNFAIP8, MAP3K14), proliferation and differentiation (IKZF1, SAE1). The LCLeQTL* transcription factor BATF interacts directly with NFKB in LCLs [43], and TNFRSF1A directly activates it [44]. The mTOR complex is activated by both LMP1 and LMP2 and enables the vast changes in energy production required for the proliferating B cells in latency III [45]. The LCLeQTL* genes regulating ATP are ADCY3, DDX6, MAPK1, ALPK2, MAP3K14, SAE1, DLEU1 and TNFAIP8 (GO annotation). Finally, the interferon pathway is dysregulated in latency III by altering expression of transcription factors such as IRF5, IRF7 and the STATs [46]. This may underpin the genetic associations with expression seen for SP140 in LCLs, so that EBV contributes to altered expression to dampen the IFN response. Several LCLeQTL*s were transcription factors of undefined roles: ZNHIT3, TBX6, RCOR1, ZFP36L2 and AFF1. These are likely to mediate some of the global changes to B cell function on infection. Also, several LCLeQTL*s had functions not readily attributable to LMP1/LMP2 signalling, and for others, their functions are largely unknown. Collectively, there is strong support for LCLeQTL* genes affecting latency III gene expression programs.

#### LCLeQTL genomic burden and EBV phenotype in vitro and in vivo

**Fig. 5:**
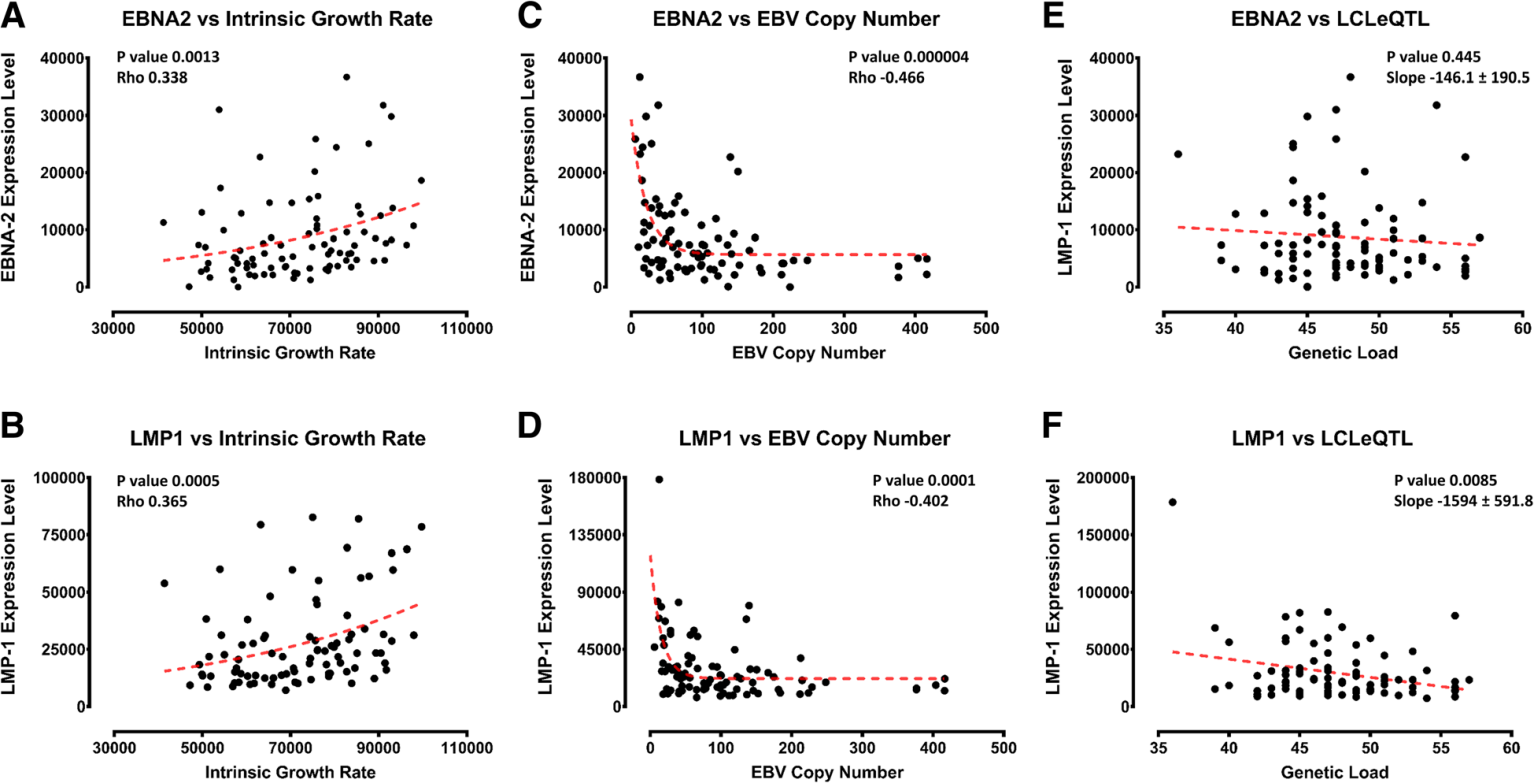
Genetic burden and EBV phenotypes. The correlation of LCL intrinsic growth rate with EBNA2 (**a**) and LMP1 (**b**) expression level in LCLs (Spearman’s rank correlation). Correlation of EBV DNA copy number with EBNA2 (**c**) and LMP1 (**d**) expression level in LCLs (Spearman’s rank correlation). Association of LCLeQTL genetic load (sum of risk alleles across all LCLeQTL SNPs) with EBNA2 (**e**) and LMP1 (**f**) expression level in LCLs (calculated using linear regression)

The effect of risk alleles on regulation of EBV infection and MS is likely to be complex, affecting LCL growth rates, states and immune responses to infected lymphocytes in vivo. We tested the hypothesis that specific risk alleles, and increasing genetic load of all LCLeQTL risk alleles, would favour increased proliferation of infected B cells. This may be detectable as increased intrinsic growth rate of LCLs [31], increased expression of EBV genes in LCLs, or increased EBV copy number in LCLs, or, albeit with many checkpoints in between, increased titre of anti EBNA1 antibodies. We first tested if each of these LCL EBV phenotypes, intrinsic growth rate, expression levels of EBV latency III genes (EBNA2, LMP1) and EBV DNA copy number in LCLs was correlated. Intrinsic growth rate was available for LCLs from 92 individuals [31]. The gene expression data for EBV from the same LCL samples was available on the EBV portal in these LCLs [32]. Also, estimated EBV DNA copy number in the same LCL samples was obtained [34]. Intrinsic growth rate and expression of EBV genes EBNA2 and LMP1 were positively correlated; EBV copy number was negatively correlated with these genes (Fig. 5). We were surprised to find an inverse relationship between intrinsic growth rate and EBV DNA copy number in LCLs (Additional file 2: Figure S4A, Additional file 1: Table S8). Overall, LCLeQTL risk allele genetic burden was slightly negatively correlated with LMP1 and EBNA2 expression (Fig. 5 E and F, Additional file 1: Table S9) and intrinsic growth rate (Additional file 2: Figure S4B, Additional file 1: Table S10). A possible explanation for this is that the risk alleles could be contributing to EBV immune evasion in a myriad of ways in vivo, so that their net effect in vitro (LCLs) may represent an artifactual balance. For example, we tested particular genes sets from our model in Fig. 4 and found most of the negative association with intrinsic growth rate/EBNA2 and LMP1 expression was due to the genes affecting energy usage (Additional file 2: Figure S4C, D). The risk alleles were associated with slower growth rate, potentially indicating a less immunogenic LCL. Alternatively, this may indicate reduced capability of the immune response independently of effect in LCLs and less control of EBV by this mechanism. Further, reduced proliferation of LCLs may correspond to an altered balance in EBV cycling, with more lytic phase production. The reverse correlation between the LCL intrinsic growth rate and EBV copy number in LCLs is consistent with this hypothesis, since EBV copy number likely increases prior to lytic phase initiation. No significant correlation was detected between genetic burden and antiEBNA1 titres, although low correlation was seen for some SNPs (Additional file 1: Table S11). Again, it is difficult to determine the significance of this, given the multiple factors affecting this parameter.

### CD40 and TRAF3 expression and risk genotype in B cells and LCLs

**Fig. 6:**
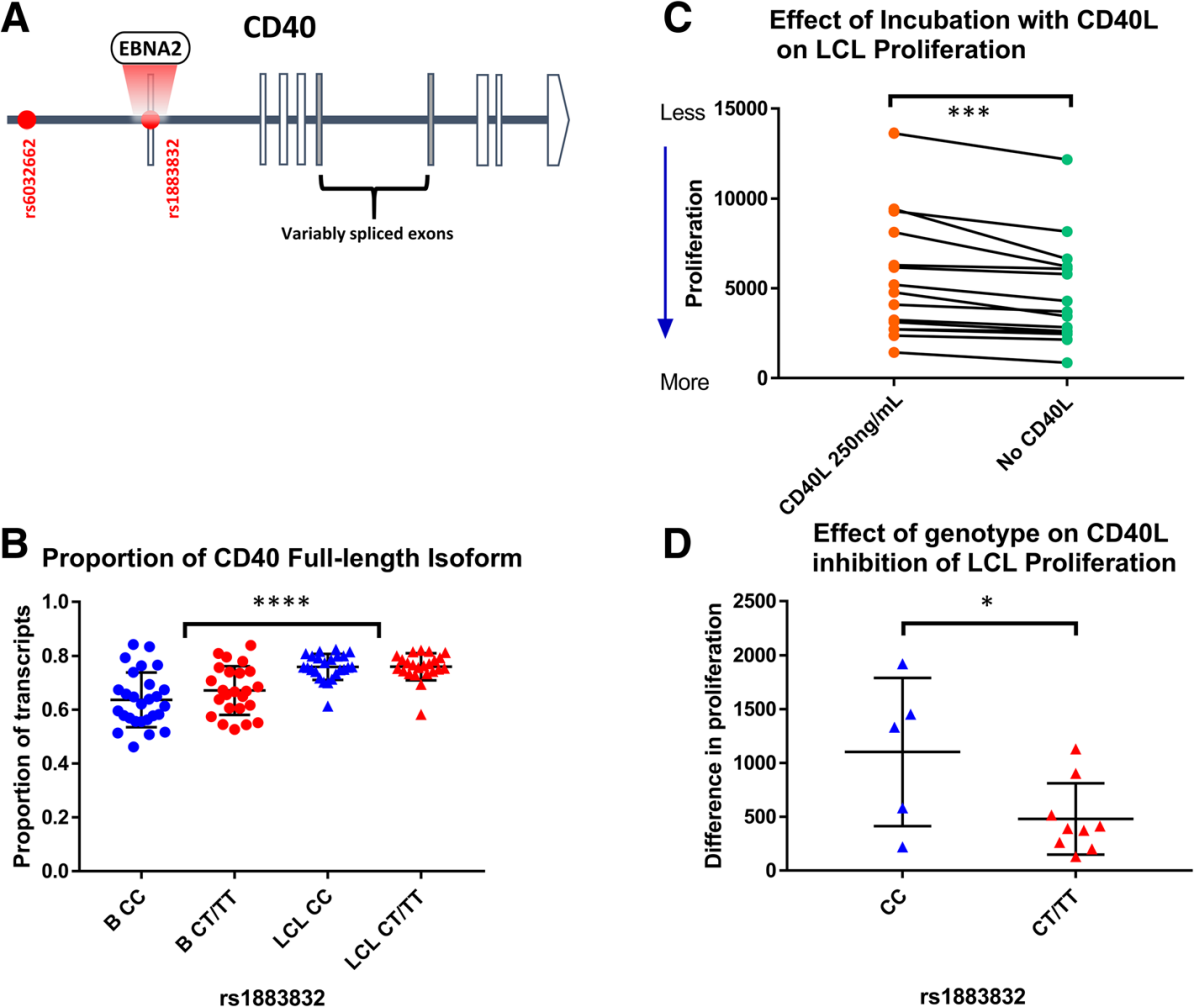
MS risk gene CD40 expression and signalling in B cells and LCLs. **a** Risk SNP rs1883832 is a binding site for the EBV transcription factor EBNA2. **b** The proportion of full length CD40 mRNA is higher in LCLs compared to CD19+ B cells, independent of rs1883832 genotype. **c** CD40L inhibits LCL proliferation (**d**) The inhibitory effect of CD40L on LCL proliferation is greater for the protective genotype (CC). Difference in proliferation (*Y* axis) is the difference in proliferation of LCLs over 5 days cultured with or without CD40L, measured by median fluorescence intensity of cell trace violet. * < 0.05,*** < 0.001, **** < 0.0001

Expression of CD40 is highest for the protective genotype (SNP rs1883832 T) in blood [19] and B cells [36]. We hypothesised this higher expression decreased signalling through LMP1 through competition for the same signalling molecules. We first confirmed that its expression was higher in LCLs in GTEx data [47] for whole blood (*n* = 396, *p* < 10^−8^, rank of *p* value 29/4566 genes) and LCLs (*n* = 117, *p* < 0.06, rank 330/4566), and in the RTeQTL data [38] (*n* = 955, combined *p* < 10^−8^ for GTEx and RTeQTL data). Earlier, we had shown that the protective allele decreases mRNA encoding secreted CD40 (sCD40, exon 6 spliced out) in B cells and dendritic cells [36]. In our LCL cohort (Westmead Institute for Medical Research; WIMR LCLs), the full-length isoforms make up 80% of transcripts, whereas they are only 65% of transcripts in B cells (*p* < 10^−9^, Fig. 6a, b, Additional file 2: Figure S5). Isoform usage was genotype independent in both cell types. We then tested if signalling through CD40 reduced LCL proliferation by culture with/without CD40L. We found that proliferation of LCLs in the presence of CD40L is decreased (*p* < 0.0001, Fig. 6c, Additional file 2: Figure S6–7), with more inhibition for the protective genotype (*p* < 0.03, Fig. 6d). This is consistent with the CD40 protective genotype effect on MS being due to reduced susceptibility to Latency III proliferation of EBV.

**Fig. 7:**
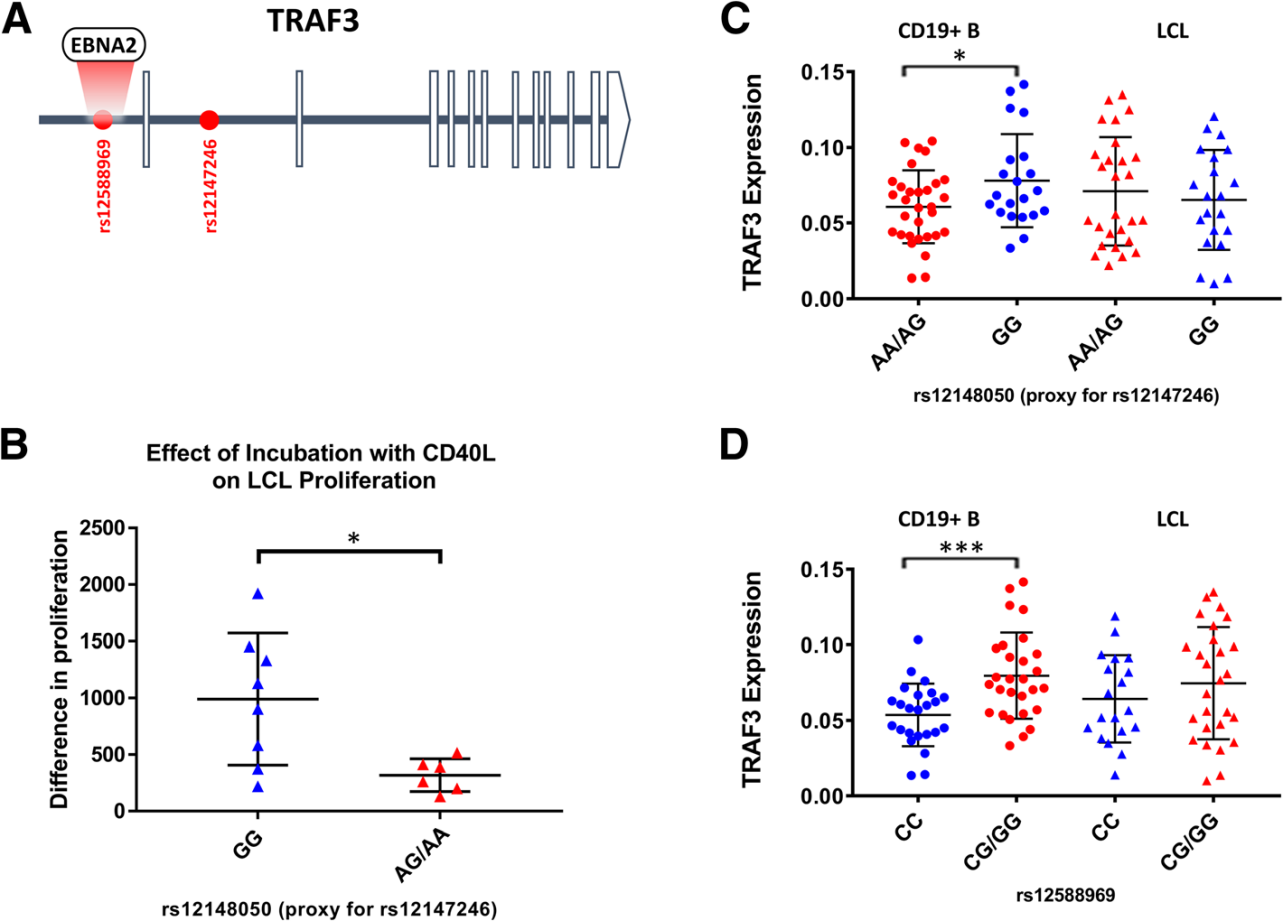
The effect of MS risk SNP genotype on the expression of TRAF3. **a** Risk SNP rs12588969 is a binding site for the EBV transcription factor EBNA2. The inhibitory effect of CD40L on LCL proliferation is greater for the protective genotype (GG) of rs1214050 (**b**). Difference in proliferation (*Y* axis) is the difference in proliferation of LCLs over 5 days cultured with or without CD40L, measured by median fluorescence intensity of cell trace violet. TRAF3 expression is genotype dependent in CD19+B cells for MS risk SNPs rs12147246 (measured by proxy SNP rs1214050) (**c**) and rs12588969 (**d**). * < 0.05, ***, < 0.001

TRAF3 is an MS risk gene with two independent association signals, for SNPs rs12588969 and rs12147246 [16]. TRAF3 protein binds to the CTAR1 domain of LMP1 and reduces signalling through TRAF6, which binds to the CTAR2 domain of LMP1 (see Fig. 4). We found that the protective variant of the risk SNP rs12588969 is associated with lower expression of TRAF3 in GTEx LCLs (*p* < 0.0001) and in WIMR B cells, with a trend in LCLs (Fig. 7), and replicated in the RTeQTL dataset (combined *p* < 10^−15^), but not in blood. This SNP is in a locus bound by the EBV transcription factor EBNA2 (Fig. 7a). The protective allele of the second SNP rs12147246 (using SNP rs12148050 as a proxy SNP) was associated with higher expression of TRAF3 in B cells and in blood, but not in GTEx or WIMR LCLs. The protective SNP of rs12148050 was associated with reduced proliferation of LCLs in the presence of CD40L (*p* = 0.02, Fig. 7d), but no association was seen for rs12588969.

These data are consistent with both CD40 and TRAF3 rs12148050 protective SNPs reducing susceptibility to EBV due to genotype effects in B cells and LCLs, conferring protection through decreasing signalling through the LMP1 pathways in newly infected B cells or LCLs. However, the expression pattern for rs12588969 is inconsistent with this. The complexity of these findings for TRAF3 may be due to different isoform usage, use of the NFKB alternative pathway, or context-dependent function in B and LCLs.

**Fig. 8:**
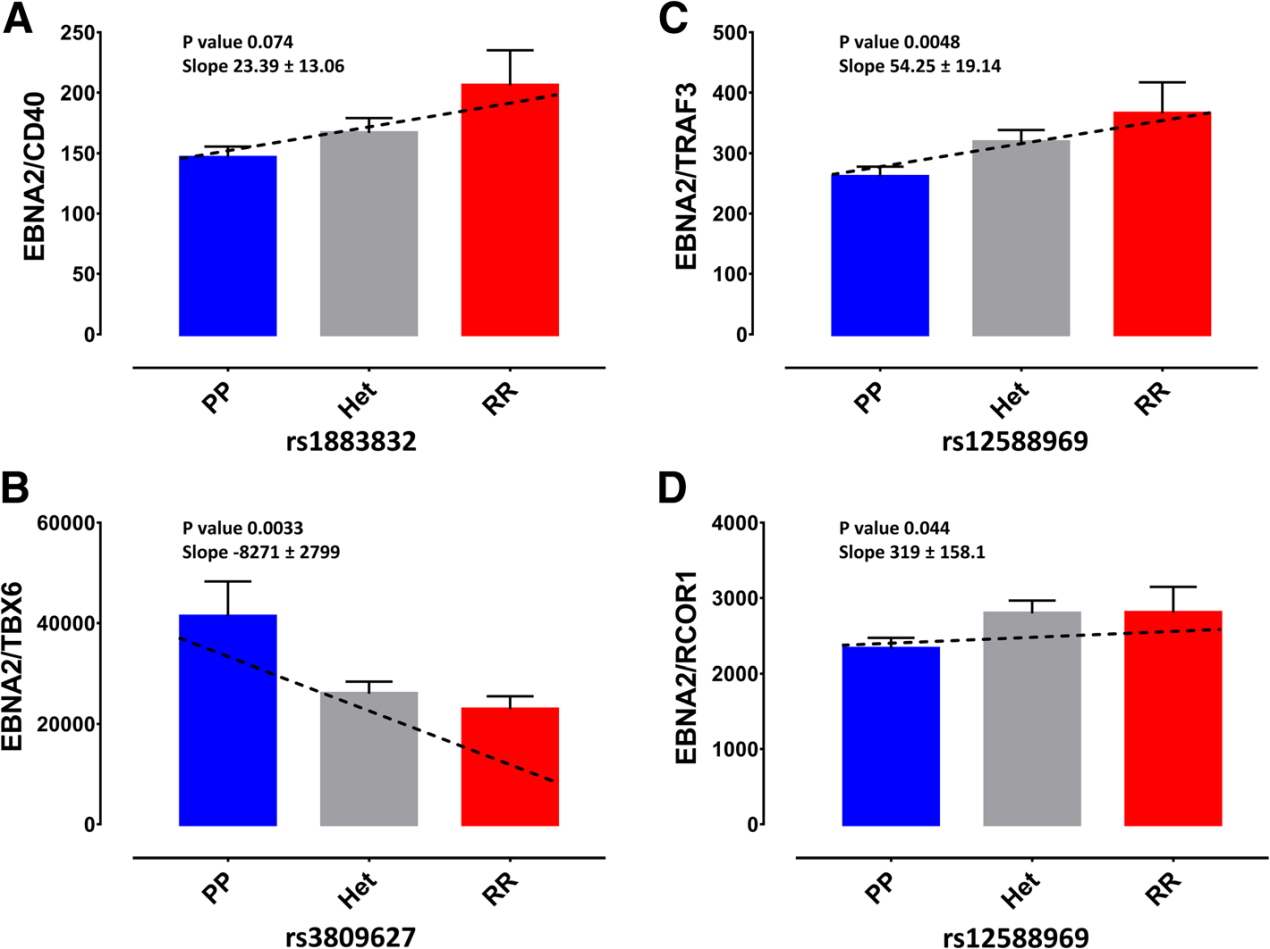
Effect of genotype on the expression ratio of EBNA2 with MS risk genes. **a** EBNA2/CD40, (**b**) EBNA2/TBX6, (**c**) EBNA2/TRAF3 and (**d**) EBNA2/RCOR1. Genotype effect on expression ratio was calculated using linear regression. Dotted line is regression slope, bars are mean + SEM. RR, homozygous risk genotype, Het, heterozygote, PP, homozygous protective genotype

Finally, TRAF3 and CD40 risk SNPs are co-localised with EBNA2 Chip-Seq peaks (Figs. 6 and 7), as are the risk SNPs for four other LCLeQTLs. Consistent with an EBNA2 effect on their expression, we now show that the expression of TRAF3, CD40, TBX6, RCOR1 and CLECL1 in LCLs is correlated with EBNA2 expression, dependent in each case on the risk/protective genotype (Fig. 8, Additional file 2: Figure S8–9). For TRAF3, those with the risk allele targeted by EBNA2 (rs12588969) expression was lower when EBNA2 expression was higher (Fig. 8b), consistent with lower expression of this gene for risk allele (Fig. 7), and resultant reduced inhibition LMP1 signalling. Similarly, the protective allele of CD40 has higher expression when EBNA2 expression is lower, consistent with higher expression inhibiting LMP1 signalling. The CD40/TRAF3 risk allele genetic load is negatively correlated with LMP1 expression (Additional file 2: Figure S10).

## Discussion

We sought evidence that response to EBV latency III infection contributed to MS susceptibility from the recently expanded list of MS risk loci [16]. The number of MS risk SNPs associated with genes differentially expressed between infected and uninfected B cells was 139 of 255, much more than would be expected by chance. The number of MS risk SNPs where the genotype was associated with proximal gene expression (44 of the 201 risk SNPs) in LCLs was also much higher than would be expected by chance. Thirty-five of these 44 were more associated with expression in LCLs than they were with blood. EBV transcription factor binding sites are over-represented among MS risk genes and among LCLeQTLs, including at risk genotype sites for six genes. Expression of five of these genes was associated with EBNA2 expression in LCLs dependent on genotype, including CD40 and TRAF3. Expression of the EBV LMP1 homologue CD40, and the LMP1 ligand TRAF3, was affected by risk genotype in EBV-infected cells and/or B cells. Infected B cell proliferation was reduced on signalling through CD40, and more so for the protective genotypes of CD40 and TRAF3. The 37 MS risk genes identified as LCLeQTL* have plausible roles in signalling on the LMP1 and LMP2 pathways in EBV latency III. The total genetic burden of LCLeQTL risk alleles was negatively correlated with intrinsic growth rate and EBV gene expression in LCLs. No correlation between genetic burden and serum antiEBNA1 antibody titres was detected.

Although these data provide genetic evidence that EBV has a facilitative role in MS, they are not conclusive. The association of MS risk genotypes with LCL expression, and the enrichment of risk genes involved in B cell proliferation and utilised by EBV, may be due to B cell processes contributing to disease, and independent of the role in EBV latency III. Notably though, we found the LCLeQTL genes were more dysregulated between LCLs and unstimulated B cells than between activated B cells and unstimulated B cells. Also, even though the interaction between CD40/TRAF3/LMP1 would predict protective genotypes decrease EBV latency III proliferation, this proliferation may be independent of the pathogenic effect in MS of these genes. Finally, the association of MS risk SNPs with expression and LCL proliferation may be different in B cells from people with MS. It should be noted that the MS risk SNPs are those affecting susceptibility to MS, so they should be associated with processes affecting pathogenesis. LCLs obtained from those who have already developed disease may have a phenotype representing disease state rather than susceptibility. Consequently, control samples may be more useful for detecting effects of risk SNPs on pathogenesis.

Genes affecting MS susceptibility favour processes leading to dysregulated immune responses, and if the processes are ongoing and drive MS pathogenesis, reversing these would be expected halt progression. Monoclonal antibodies to risk gene IL2Ra (drug Daclizumab) and to CD49D, ligand of risk gene VLA4 (drug Natalizumab), are effective therapies for MS [2]. More generally, drugs which remove immune cells expressing risk genes, such as antiCD20 (B cells) and antiCD52 (Alemtuzumab), or which corral them in secondary lymphoid organs (Fingolimod) are also effective.

Similarly, if poor regulation of EBV infection contributes to MS susceptibility and progression via processes tagged by the MS risk gene LCLeQTLs, therapeutic strategies to favour immune control of EBV may halt or slow progression. Especially attractive are targets on the EBV genome, since these greatly increase specificity for EBV compared to the host genes which regulate LMP1 and LMP2 signalling. Of these EBV targets, methods of reducing EBNA2 expression or activation through its binding partners are in development for other conditions. Farrell et al. [48] have shown a cell-permeable peptide inhibiting EBNA2 binding to co-transcription factor CBF1 results in downregulation of EBV proteins LMP1 and LMP2 and reduced LCL proliferation. EBNA2 and other EBV transcription factors could be inactivated with complementary nucleic acid therapeutics, as currently used for familial amyloidotic polyneuropathy [49].

Although it is challenging to prove the MS risk genotype is associated with MS because it alters Latency III proliferation and immune evasion, further support would come from demonstrating [1] that reduction of EBNA2 and targeted EBV (B95.8 strain) miRNAs reduce the genotype association with expression in LCLs [2]; altered expression of the risk gene corresponding to the LCLeQTL effect alters LCL proliferation, especially through the pathway the gene affects; and [3] effects may be exaggerated in LCLs derived from MS patients. Testing of genotype effects on expression and function in other EBV phases is also warranted. Finally, the killing of LCLs by EBV-specific T cells or NK cells in co-cultures may be risk genotype and gene dependent.

## Conclusions

These data indicate many genetic risk factors identified in genome wide association studies for MS susceptibility have roles consistent with a dysregulated response to EBV infection, and so in this way contribute to MS pathogenesis. They point to particular molecular processes important in regulating LCL proliferation, and so molecular targets for control of EBV infection to potentially reduce MS progression. Specifically, these data indicate targeting EBV EBNA2, and MS risk genes functioning on the LMP1/2 pathways, and the pathways themselves, may be of therapeutic benefit in MS.

## Additional files

Additional file 1: **Table S1**. Genes dysregulated due to EBV infection. **Table S2.** LCLeQTL gene list. **Table S3.** Replication and combined *p* value for LCLeQTL gene list. **Table S4.** LCLeQTL* gene list. **Table S5.** Whole blood eQTLs gene list. **Table S6.** Whole blood eQTLs minus LCLeQTL*. **Table S7.** EBV Transcription Factor interactome gene list. **Table S8.** Intrinsic growth rate and genetic load of risk alleles. **Table S9.** Expression of EBV genes and genetic load of risk alleles. **Table S10.** EBV copy number in LCLs and genetic load of risk alleles. **Table S11.** LCLeQTL SNP sets and EBNA1 titres. (XLSX 730 kb)

Additional file 2: **Figure S1.** eQTL data of MS risk variants in whole blood (A) and LCLs (B). **Figure S2.** Identifying gene expression associated with MS risk SNPs in LCL and WB context. **Figure S3.** Identifying EBV transcription factors binding peak overlap with MS risk SNPs and SNPs in LD with them workflow. **Figure S4.** (A) Spearman’s correlation between intrinsic growth rate and EBV copy number in LCLs, (B) Association between intrinsic growth rate and genetic load of risk alleles of LCLeQTL (calculated using linear regression), (C) association between LMP1 expression level and energy production-related genes in LMP1 signalling pathway genes (calculated using linear regression), (D) correlation between EBNA2 expression level and energy production-related genes in LMP1 signalling pathway genes (calculated using linear regression). Genetic load refers to the sum of the risk alleles for each set of SNPs tested. **Figure S5.** CD40 isoforms in B cells and LCLs for CD40 MS risk SNP rs188383. **Figure S6.** LCL Survival on CD40 ligand treatment. **Figure S7.** Cell trace violet dilution on CD40L stimulation for CD40 rs1883832 genotype. **Figure S8.** Effect of genotype on the expression ratio of EBNA2 with MS risk genes. (A) EBNA2/CLECL1, (B) EBNA2/TNFRSF1A, (C) EBNA2/TNFAIP8. **Figure S9.** The correlation between EBNA2 and expression level for MS risk genes CD40, TRAF3 and CLECL1, where risk SNP is co-located in EBNA2 binding peaks. **Figure S10.** LMP-1 expression level and genetic load of CD40 and TRAF3 risk alleles. (PDF 1470 kb)

## Abbreviations

EBV: Epstein-Barr virus
eQTL: Expression quantitative trait loci
GWAS: genome wide association study
LCL: Lymphoblastoid cell lines
MS: Multiple sclerosis
TF: Transcription factor
