# Supplementary material for: Evidence from genome wide association studies implicates reduced control of Epstein-Barr virus infection in multiple sclerosis susceptibility
Source: Genome Med. 2019 Apr 30;11:26. doi: 10.1186/s13073-019-0640-z (PMC6492329; doi:10.1186/s13073-019-0640-z)
Supplement: Supplementary file 2 — Figure S1. eQTL data of MS risk variants in whole blood (A) and LCLs (B). Figure S2. Identifying gene expression associated with MS risk SNPs in LCL and WB context. Figure S3. Identifying EBV transcription factors binding peak overlap with MS risk SNPs and SNPs in LD with them workflow. Figure S4. (A) Spearman’s correlation between intrinsic growth rate and EBV copy number in LCLs, (B) Association between intrinsic growth rate and genetic load of risk alleles of LCLeQTL (calculated using linear regression), (C) association between LMP1 expression level and energy production-related genes in LMP1 signalling pathway genes (calculated using linear regression), (D) correlation between EBNA2 expression level and energy production-related genes in LMP1 signalling pathway genes (calculated using linear regression). Genetic load refers to the sum of the risk alleles for each set of SNPs tested. Figure S5. CD40 isoforms in B cells and LCLs for CD40 MS risk SNP rs188383. Figure S6. LCL Survival on CD40 ligand treatment. Figure S7. Cell trace violet dilution on CD40L stimulation for CD40 rs1883832 genotype. Figure S8. Effect of genotype on the expression ratio of EBNA2 with MS risk genes. (A) EBNA2/CLECL1, (B) EBNA2/TNFRSF1A, (C) EBNA2/TNFAIP8. Figure S9. The correlation between EBNA2 and expression level for MS risk genes CD40, TRAF3 and CLECL1, where risk SNP is co-located in EBNA2 binding peaks. Figure S10. LMP-1 expression level and genetic load of CD40 and TRAF3 risk alleles. (PDF 1470 kb) [file 13073_2019_640_MOESM2_ESM.pdf]

Additional file 2

Supplementary Figures

Fig. S1. eQTL data of MS risk variants in whole blood (A) and LCLs (B).

Fig. S2. Identifying gene expression associated with MS risk SNPs in LCL and WB context.

Fig. S3. Identifying EBV Transcription factors binding peak overlap with MS risk SNPs and SNPs in LD with them workflow.

Fig. S4. A) Spearman's correlation between Intrinsic growth rate and EBV copy number in LCLs, B) Association between Intrinsic growth rate and genetic load of risk alleles of LCLeQTL (calculated using linear regression), C) association between LMP1 expression level and energy production related genes in LMP1 signalling pathway genes (calculated using linear regression), D) correlation between EBNA2 expression level and energy production related genes in LMP1 signalling pathway genes (calculated using linear regression). Genetic load refers to the sum of the risk alleles for each set of SNPs tested.

Fig. S5. CD40 isoforms in B cells and LCLs for CD40 MS risk SNP rs188383

Fig. S6. LCL Survival on CD40 ligand treatment

Fig. S7. Cell trace violet dilution on CD40L stimulation for CD40 rs1883832 genotype

Fig. S8. Effect of genotype on the expression ratio of EBNA2 with MS risk genes. (A) EBNA2/CLECL1, (B) EBNA2/TNFRSF1A, (C) EBNA2/TNFAIP8.

Fig. S9. The correlation between EBNA2 and expression level for MS risk genes CD40, TRAF3, CLECL1, where risk SNP is co-located in EBNA2 binding peaks.

Fig. S10. LMP-1 expression level and genetic load of CD40 and TRAF3 risk alleles.

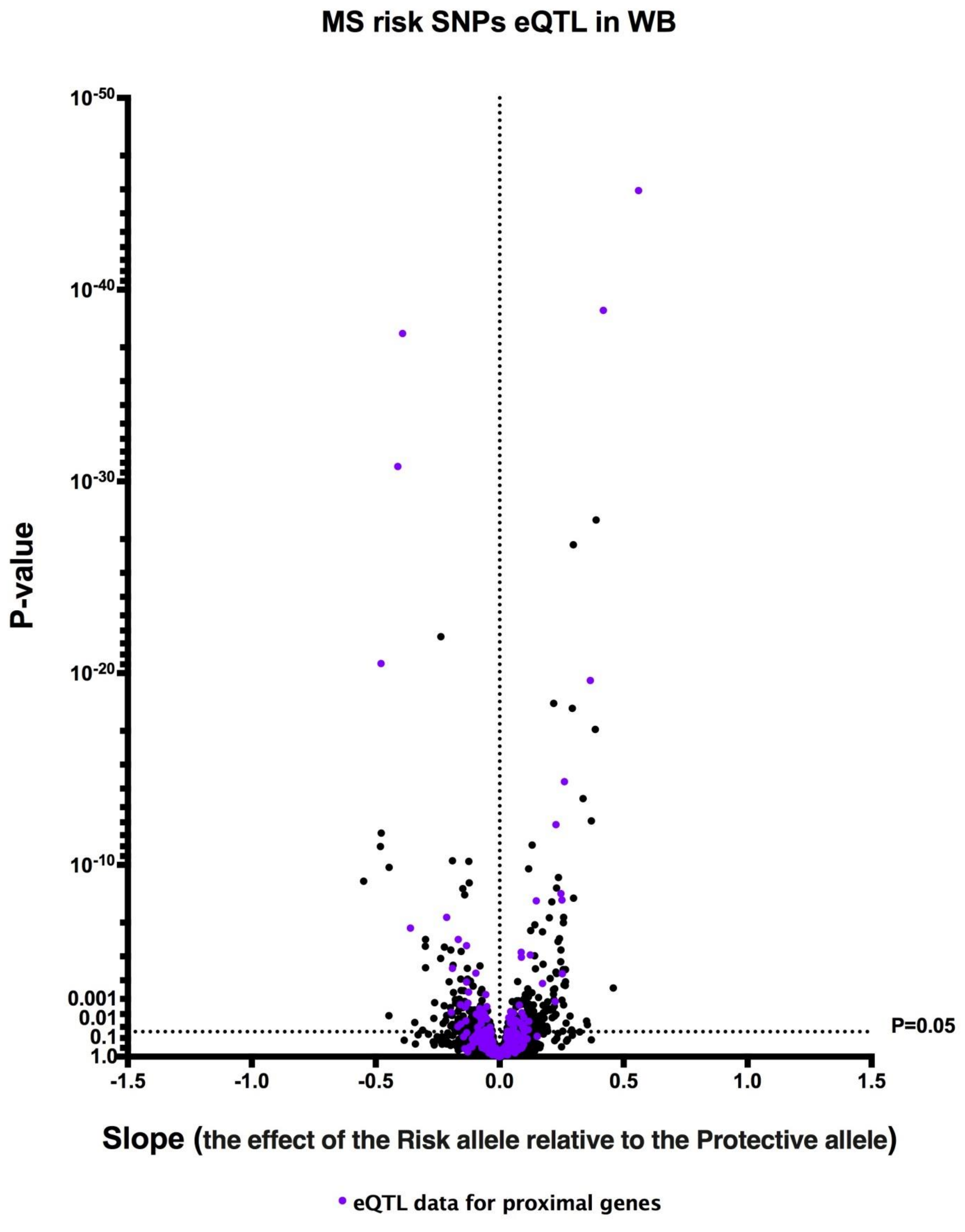

Fig. S1A. eQTL data of MS risk variants in whole blood. The volcano plot shows the significance of all eQTL data associated with MS risk SNPs in whole blood (WB) which are extracted from GTEx V7. Dots represent the eQTL data of individual MS risk SNP and Gene pair (SNP:Gene). Purple dots show eQTL data for proximal genes.

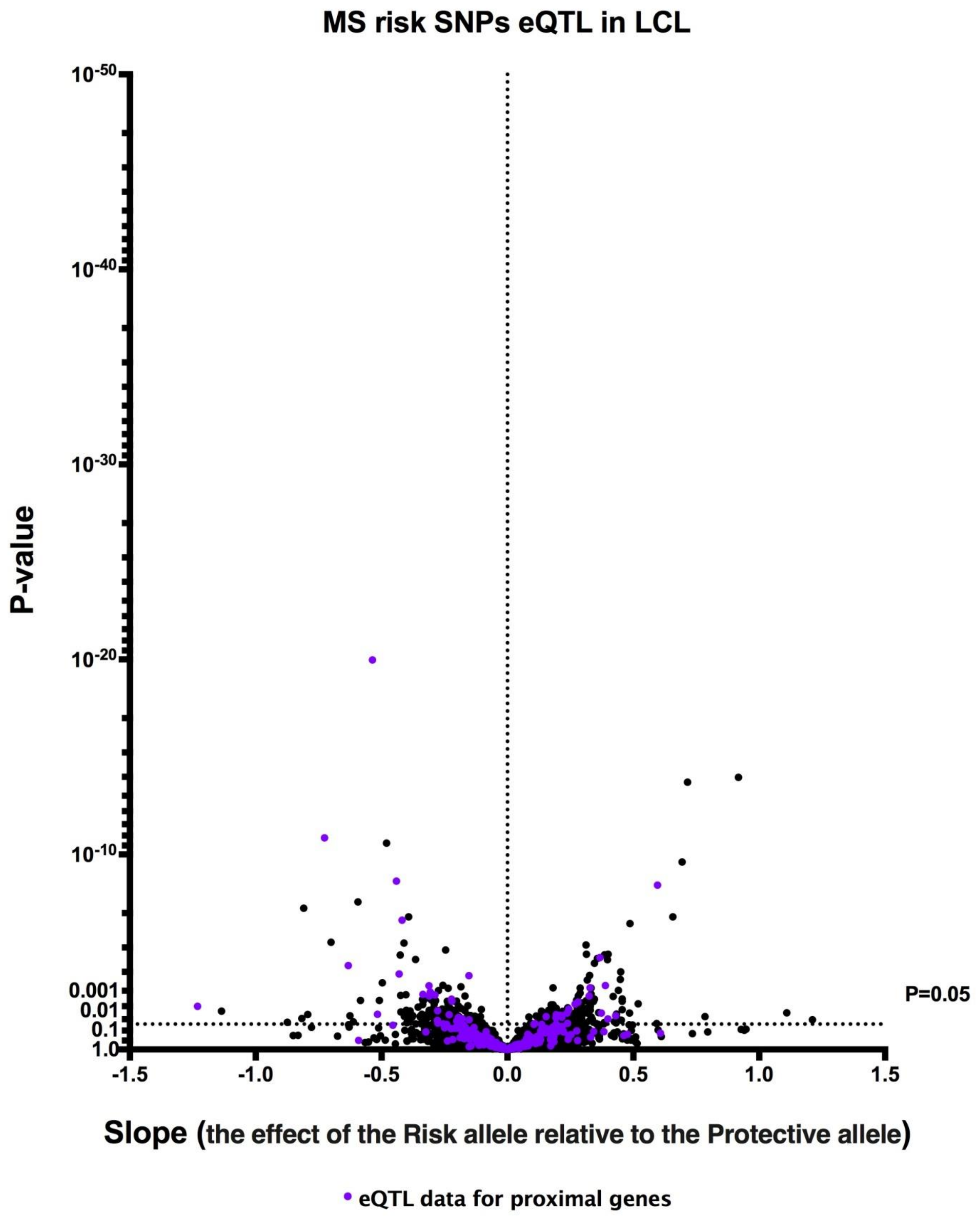

Fig. S1B. eQTL data of MS risk variants in LCL. The volcano plot shows the significance of all eQTL data associated with MS risk SNPs in EBV infected B cell (LCL) which are extracted from GTEx V7. Dots represent the eQTL data of individual MS risk SNP and Gene pair (SNP:Gene). Purple dots show eQTL data for proximal genes.

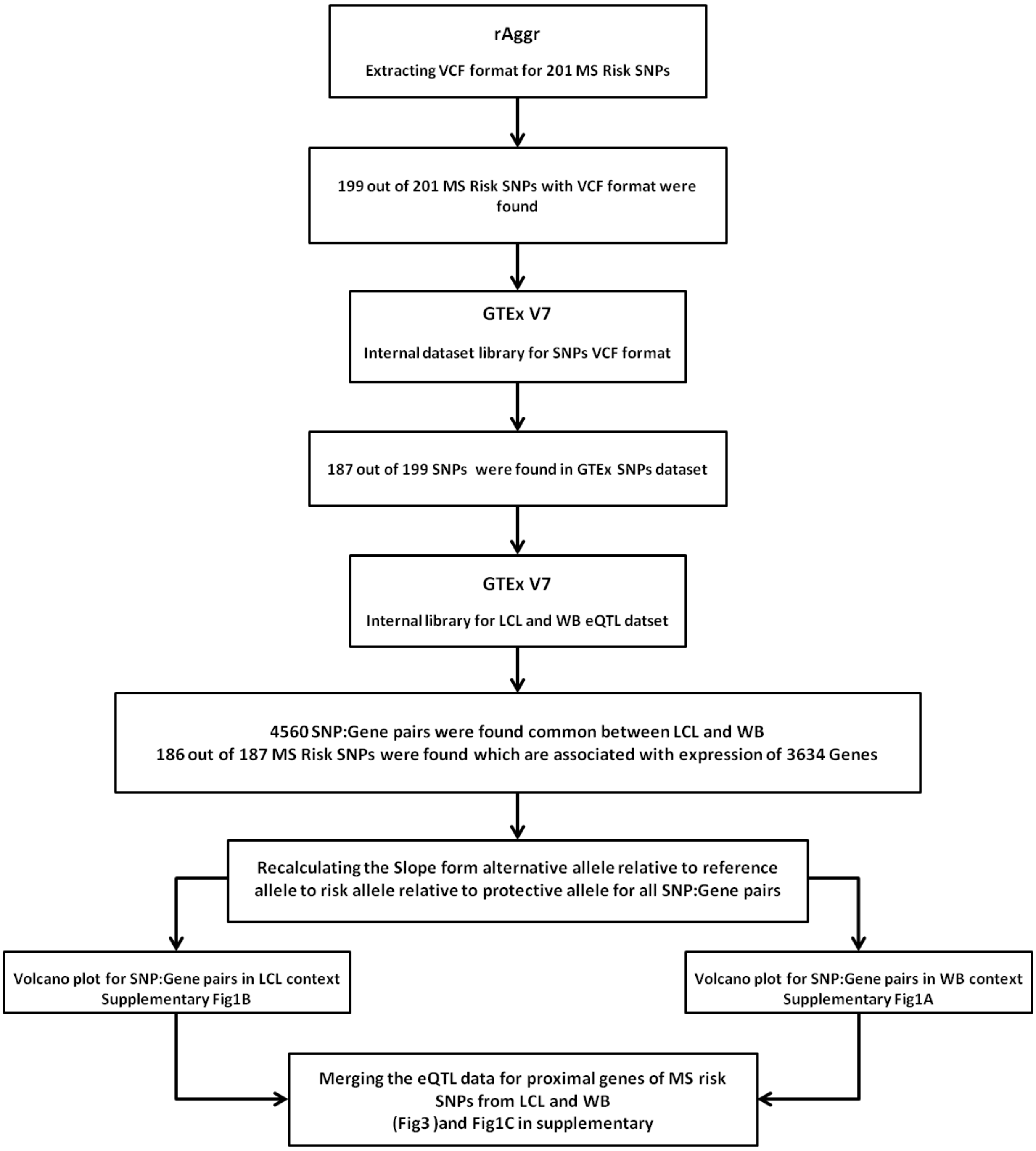

Fig. S2. Identifying gene expression associated with MS risk SNPs in LCL and WB context. The workflow shows all steps for Identifying gene expression associated with MS risk SNPs in LCL and WB context. The VCF format was identified for 201 MS risk SNPs by using rAggr and filtered by SNPs which are existed in internal library of GTEx V7. Then, all common eQTL associated with MS risk SNPs in LCL and WB were extracted from internal library of GTEx. The, proximal genes eQTL from LCL and WB were combined together to generating the final output.

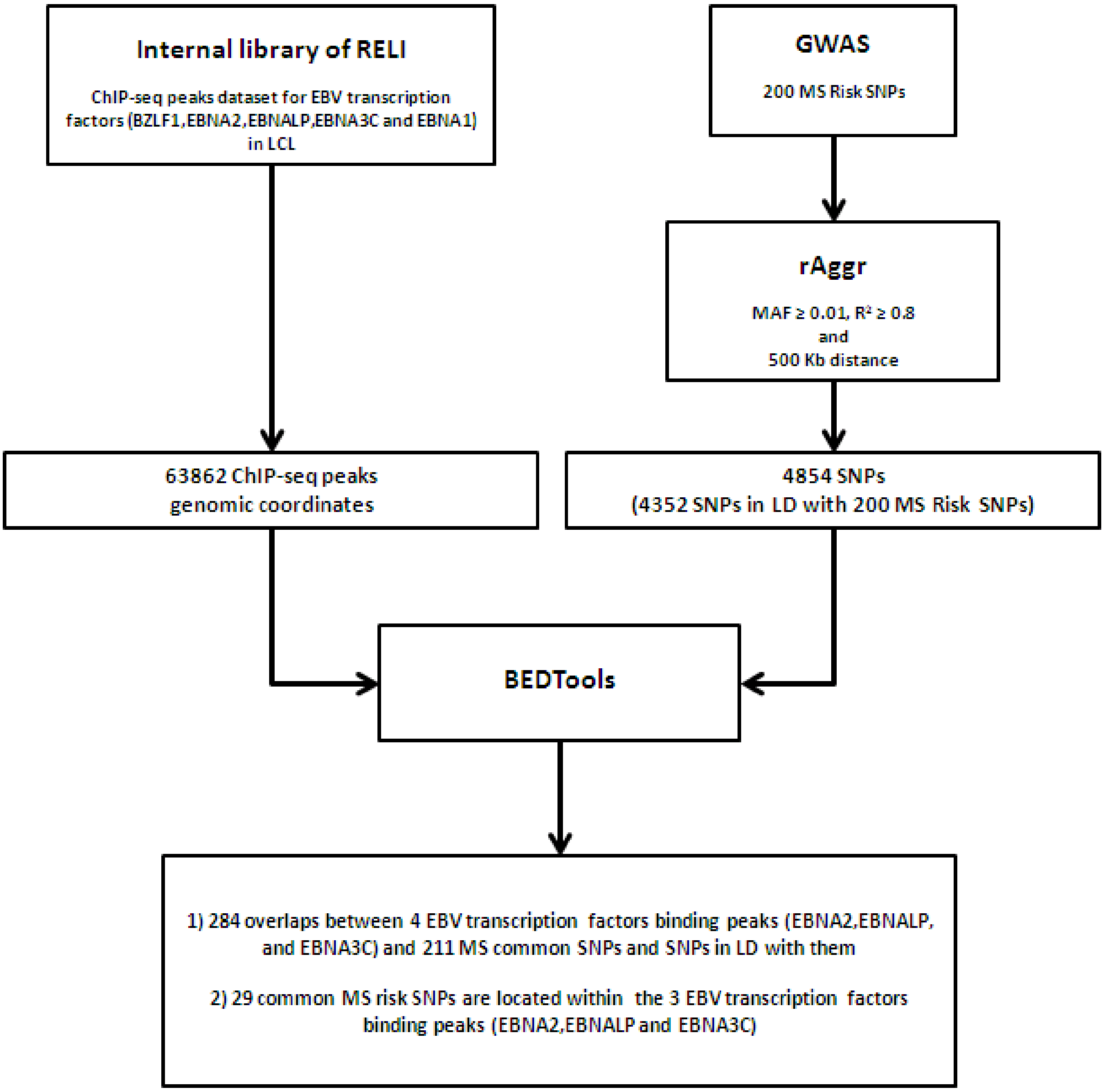

Fig. S3. Identifying EBV Transcription factors binding peak overlap with MS risk SNPs and SNPs in LD with them workflow. The workflow shows the all steps for Identifying EBV transcription factors binding peaks overlap with MS risk SNPs and SNPs in LD with them. We used internal library of Regulatory Element Local Intersection (RELI) tool to extract all genomic coordinates of EBV transcription factors binding peaks that have reported by CHIP-seq. Then we calculated the SNPs in LD with MS risk SNPs by using rAggr tool. Finally, we sought for the overlap between genomic coordinates of EBV transcription factors binding peaks and MS risk SNPs and SNPs in LD with them by using BEDTools software.

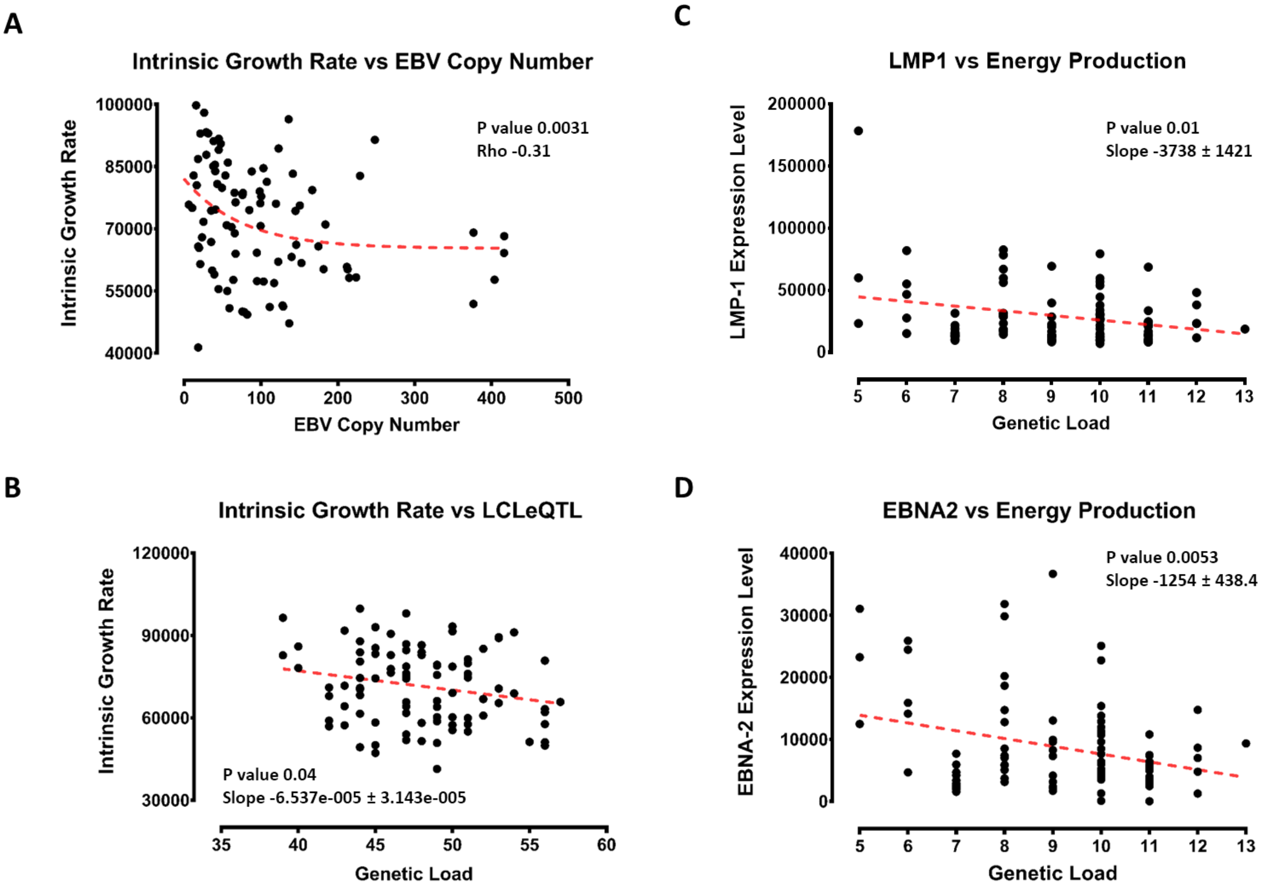

Fig. S4. A) Spearman's correlation between Intrinsic growth rate and EBV copy number in LCLs, B) Association between Intrinsic growth rate and genetic load of risk alleles of LCLeQTL (calculated using linear regression), C) association between LMP1 expression level and energy production related genes in LMP1 signalling pathway genes (calculated using linear regression), D) correlation between EBNA2 expression level and energy production related genes in LMP1 signalling pathway genes (calculated using linear regression). Genetic load refers to the sum of the risk alleles for each set of SNPs tested.

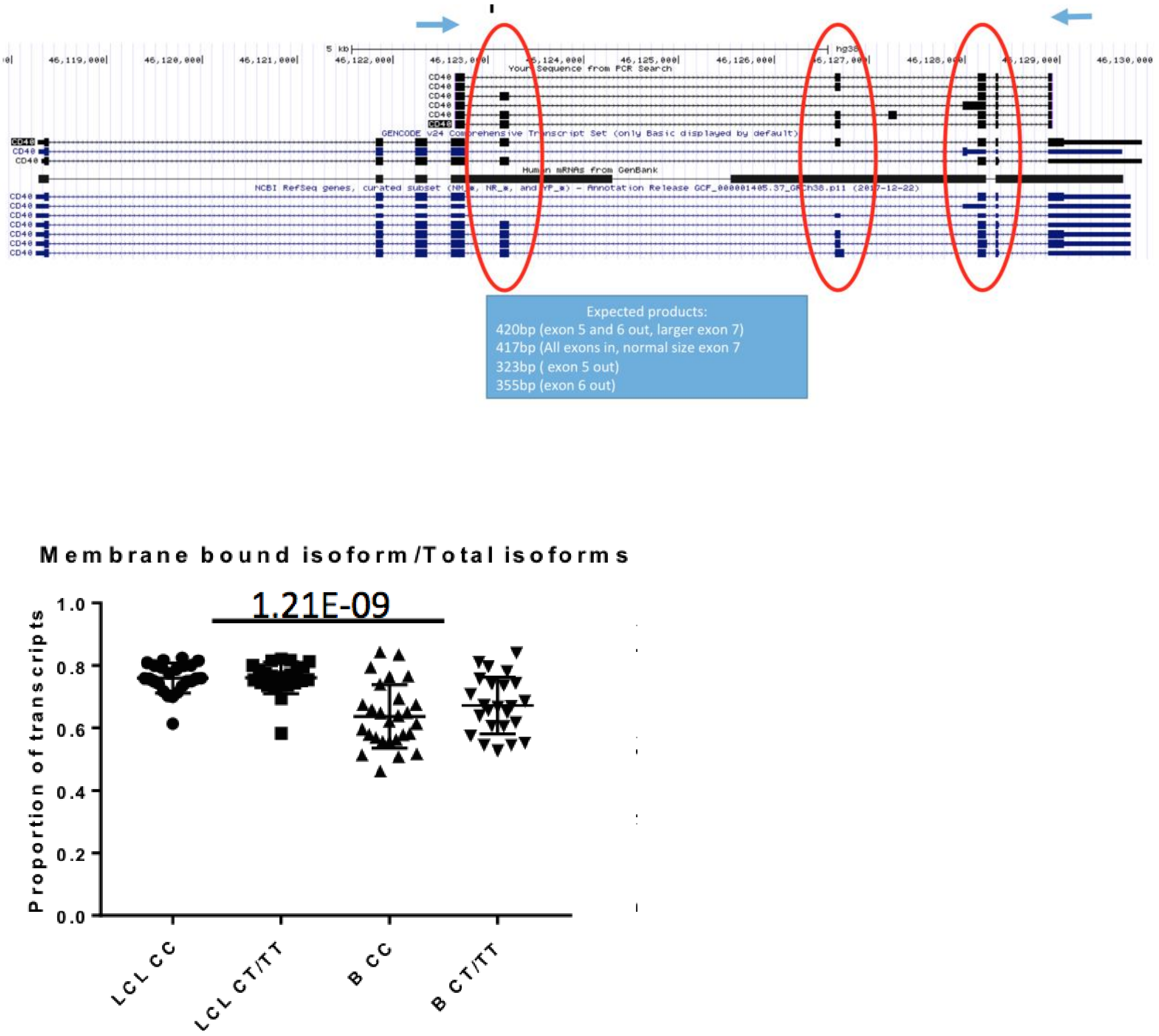

Fig. S5. CD40 isoforms in B cells and LCLs for CD40 MS risk SNP rs188383. Blue arrows indicate location of PCR primers used to amplify the different CD40 isoforms. Proportion of isoform amplified using these primers was determined by running PCR products on a Agilent Tapestation and calculation of relative molarity of each different size product.

A

B

C

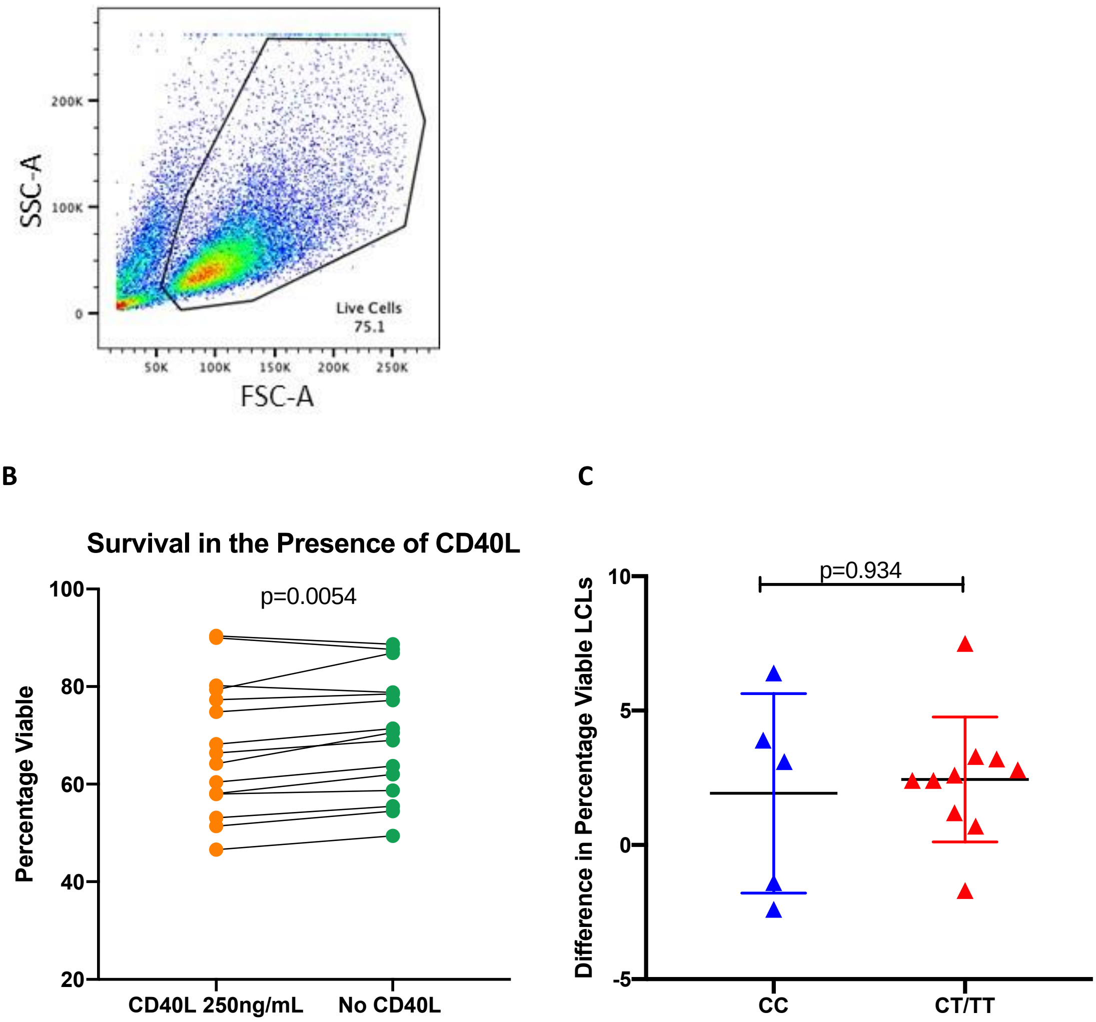

Fig. S6. LCL Survival on CD40ligand treatment. (A) Representative plot of forward scatter (FSC) vs side scatter (SSC) of LCLs at day 5. (B) CD40L significantly decreases survival of LCLs over 5 days. (C) Difference in viability between CD40L treated and control LCLs over 5 days is not significant according to genotype.

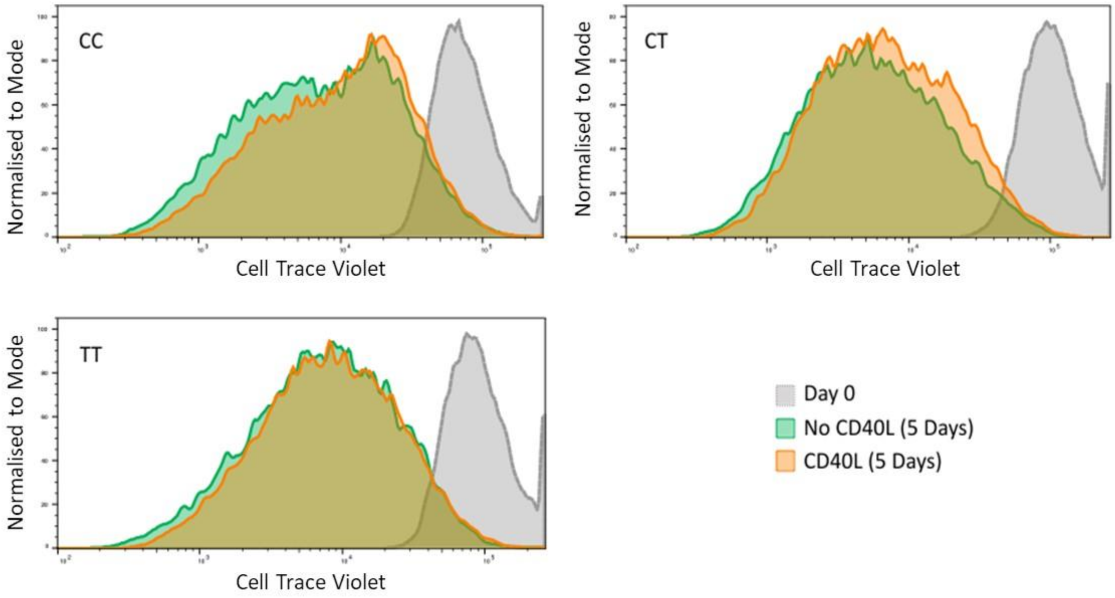

Fig. S7. Histograms comparing proliferation of LCLs with or without CD40L stimulation using Cell Trace Violet dilution. Representative plots of each CD40 rs1883832 genotype indicated.

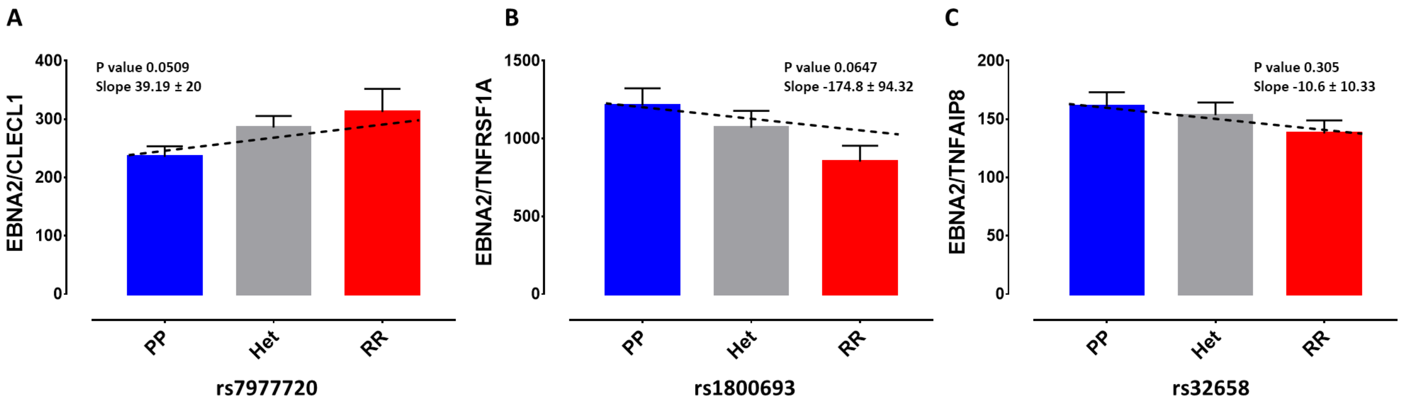

Fig. S8. Effect of genotype on the expression ratio of EBNA2 with MS risk genes. (A) EBNA2/CLECL1, (B) EBNA2/TNFRSF1A, (C) EBNA2/TNFAIP8. Genotype effect on expression ratio was calculated using linear regression. RR, homozygous risk genotype, Het, heterozygote, PP, homozygous protective genotype.

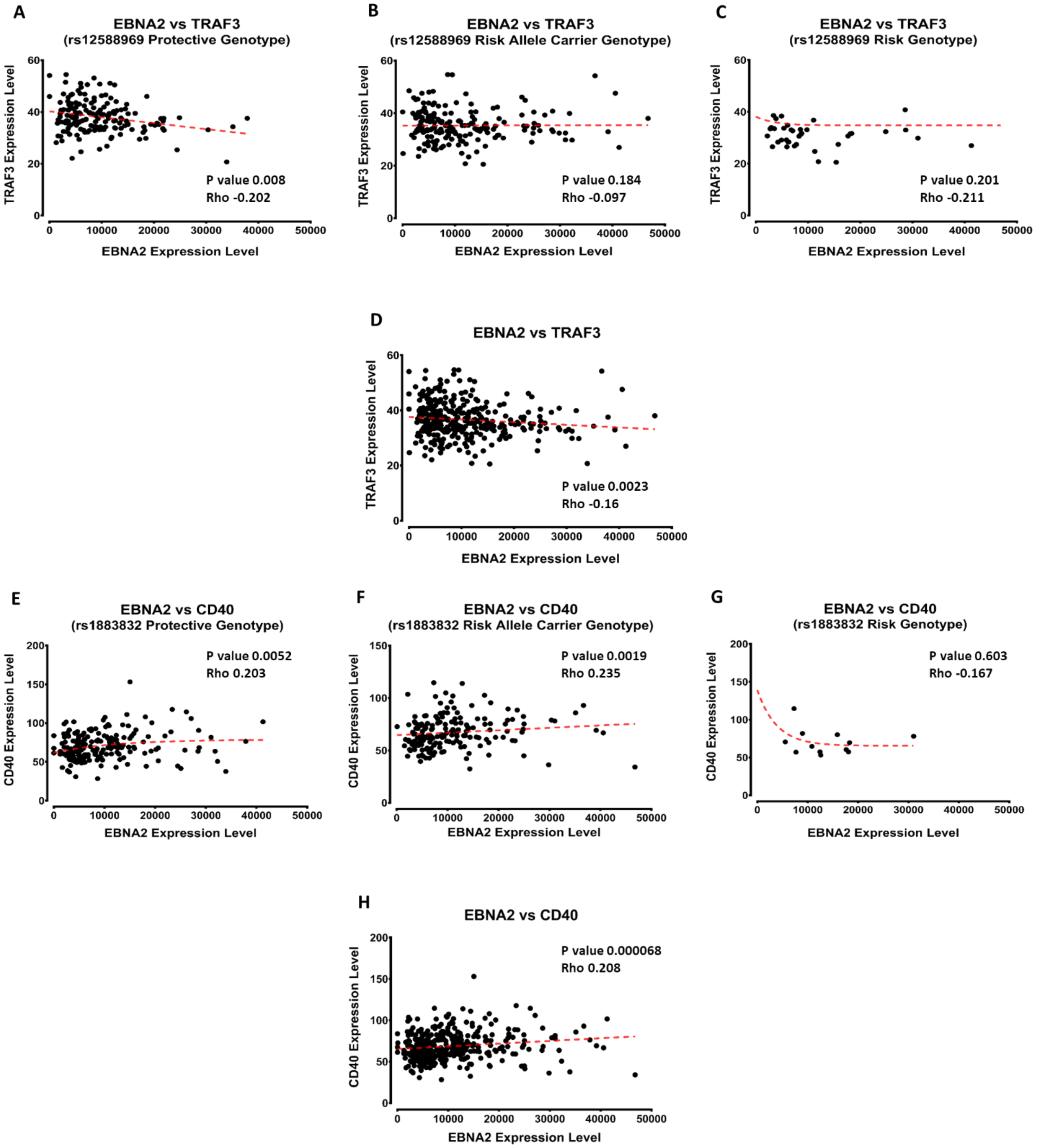

Fig. S9. The correlation between EBNA2 and expression level for MS risk genes CD40, TRAF3, CLECL1, where risk SNP is co-located in EBNA2 binding peaks. A, B, C and D indicate the correlation of EBNA2 with TRAF3 when rs12588969 (TRAF3's proximal MS risk SNP) is protective genotype, risk allele carrier, risk genotype and all samples without considering the genotype, respectively. E, F, G and H indicate the correlation of EBNA2 with CD40 when rs1883832 (CD40's proximal MS risk SNP) is protective genotype, risk allele carrier, risk genotype and all samples without considering the genotype, respectively. I, J, K and L indicate the correlation of EBNA2 with CLECL1 when rs7977720 (MS risk SNP which is proximal to CLECL1) is protective genotype, risk allele carrier, risk genotype and all samples without considering the genotype, respectively. Gene expression and genotype data extracted from 1000 genome project. All correlations tested using Spearman Rank-Order Correlation Coefficient.

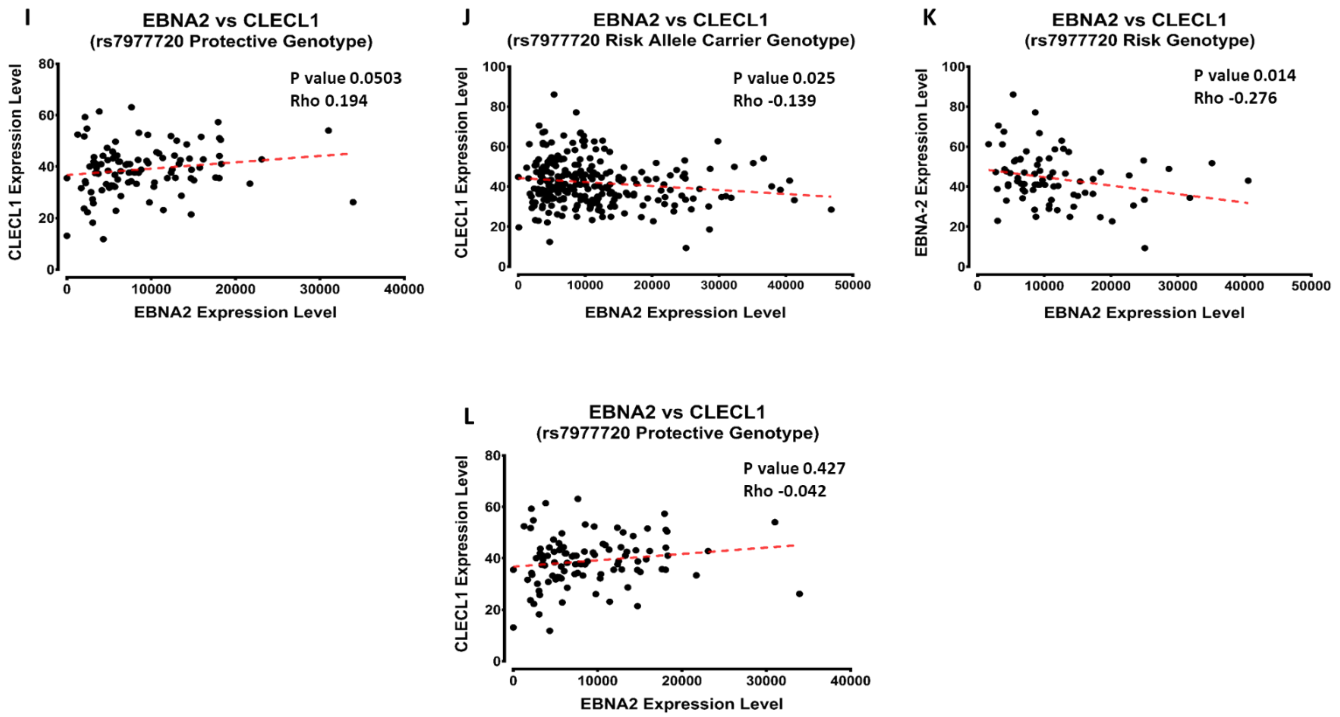

Fig. S9 (cont). The correlation between EBNA2 and expression level for MS risk genes where risk SNP is co-located in EBNA2 binding peaks. I, J, K and L indicate the correlation of EBNA2 with CLECL1 when rs7977720 (MS risk SNP which is proximal to CLECL1) is protective genotype, risk allele carrier, risk genotype and all samples without considering the genotype, respectively. Gene expression and genotype data extracted from 1000 genome project. All correlations tested using Spearman Rank-Order Correlation Coefficient.

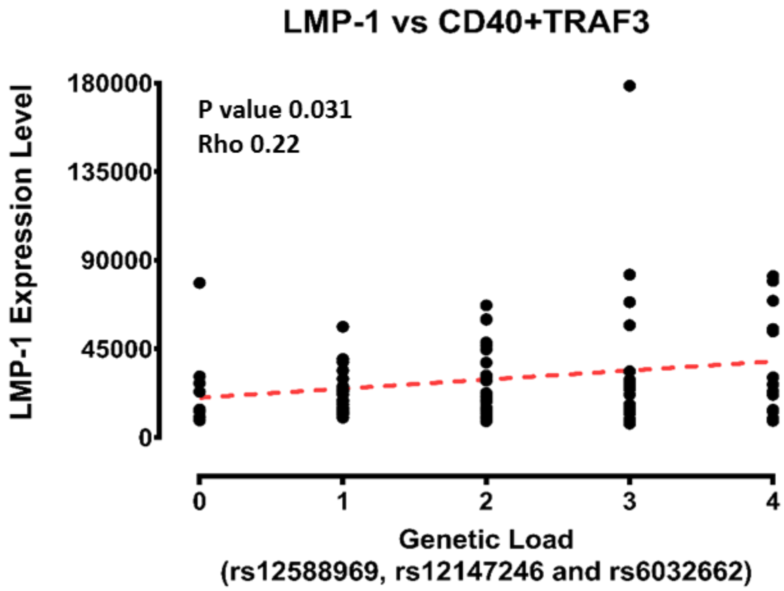

Fig. S10. LMP-1 expression level and genetic load of proximal MS risk SNPs to CD40 and TRAF3 (calculated using linear regression). Genetic load refers to the sum of the risk alleles.
